# The Gut as Reservoir of Antibiotic Resistance: Microbial Diversity of Tetracycline Resistance in Mother and Infant

**DOI:** 10.1371/journal.pone.0021644

**Published:** 2011-06-28

**Authors:** Lisbeth E. de Vries, Yvonne Vallès, Yvonne Agersø, Parag A. Vaishampayan, Andrea García-Montaner, Jennifer V. Kuehl, Henrik Christensen, Miriam Barlow, M. Pilar Francino

**Affiliations:** 1 Department of Veterinary Disease Biology, University of Copenhagen, Frederiksberg, Denmark; 2 National Food Institute, Technical University of Denmark, Lyngby, Denmark; 3 Unitat Mixta d'Investigació en Genòmica i Salut, Centre Superior d'Investigació en Salut Pública/Universitat de València-Institut Cavanilles, València, Spain; 4 Evolutionary Genomics Program, Department of Energy Joint Genome Institute, Walnut Creek, California, United States of America; 5 School of Natural Sciences, University of California Merced, Merced, California, United States of America; Argonne National Laboratory, United States of America

## Abstract

The microbiota in the human gastrointestinal tract (GIT) is highly exposed to antibiotics, and may be an important reservoir of resistant strains and transferable resistance genes. Maternal GIT strains can be transmitted to the offspring, and resistances could be acquired from birth. This is a case study using a metagenomic approach to determine the diversity of microorganisms conferring tetracycline resistance (Tc^r^) in the guts of a healthy mother-infant pair one month after childbirth, and to investigate the potential for horizontal transfer and maternal transmission of Tc^r^ genes. Fecal fosmid libraries were functionally screened for Tc^r^, and further PCR-screened for specific Tc^r^ genes. Tc^r^ fosmid inserts were sequenced at both ends to establish bacterial diversity. Mother and infant libraries contained Tc^r^, although encoded by different genes and organisms. Tc^r^ organisms in the mother consisted mainly of Firmicutes and Bacteroidetes, and the main gene detected was *tet*(O), although *tet*(W) and *tet*(X) were also found. Identical Tc^r^ gene sequences were present in different bacterial families and even phyla, which may indicate horizontal transfer within the maternal GIT. In the infant library, Tc^r^ was present exclusively in streptococci carrying *tet*(M), *tet*(L) and *erm*(T) within a novel composite transposon, Tn*6079*. This transposon belongs to a family of broad host range conjugative elements, implying a potential for the joint spread of tetracycline and erythromycin resistance within the infant's gut. In addition, although not found in the infant metagenomic library, *tet*(O) and *tet*(W) could be detected in the uncloned DNA purified from the infant fecal sample. This is the first study to reveal the diversity of Tc^r^ bacteria in the human gut, to detect a likely transmission of antibiotic resistance from mother to infant GITs and to indicate the possible occurrence of gene transfers among distantly related bacteria coinhabiting the GIT of the same individual.

## Introduction

The human gastrointestinal tract (GIT) is host to a very dense microbiota, harboring 10^13^–10^14^ bacterial cells in adults and a broad diversity of bacterial species, of which a large proportion are not yet cultured. This microbiota is often exposed to a variety of antibiotics, both directly and indirectly, due to their routine use in clinical settings and in farm animals. Therefore, its many other fundamental roles in health notwithstanding [1]–[5], the GIT microbiota may serve as an important reservoir of antibiotic resistant strains that could act as opportunistic pathogens or as donors of resistance genes to other bacteria [6]. In infants, infections due to antibiotic resistant strains are on the rise and represent a major cause of mortality and morbidity worldwide. Although the infant's gut is thought to be mostly germ-free at birth, it rapidly enters an extensive and complex process of colonization by a variety of microbes [7], [8], and recent studies have firmly established that strains from the mother's GIT can be transmitted to the infant and persist during the first weeks of life [9]. Consequently, antibiotic resistances could be vertically transmitted from the maternal GIT and bear on infant health from a very early age.

Tetracyclines are one of the most widely used groups of antibiotics worldwide and tetracycline resistance (Tc^r^) is extremely common among bacteria [10]. Presently, 43 distinct Tc^r^ genes are known and they are usually associated with large mobile genetic elements (MGE) (http://faculty.washington.edu/marilynr/). The most common forms are the active efflux of tetracycline from the cell and the synthesis of ribosomal protection proteins that prevent the binding of tetracycline to the ribosomes [10], [11]. Although its medical applications have decreased in the last decade and it is no longer used for treatment of pregnant women or children under the age of 8 years [12], tetracycline is still widely used for therapeutic treatment in animal production and in some countries it is also used as growth promoter in animal feed [11], [13]. Therefore intestinal bacteria are still extensively exposed to this antibiotic.

A recent microarray-based study has found *tet*(M) and *tet*(W) to be the most prevalent Tc^r^ genes for the oral and fecal metagenomes of healthy adults, respectively [14]. Furthermore, Tc^r^ genes like *tet*(M), *tet*(O) and *tet*(W) have also been detected in fecal samples from healthy and exclusively breast-fed infants, suggesting that Tc^r^ genes are common in the environment [15]. However, these studies have not revealed the types of bacteria that harbor these resistances in the GIT and have not addressed the potential origin of the Tc^r^ genes and strains present in healthy infants. Here, we have used a culture-independent approach to characterize the diversity of microorganisms conferring Tc^r^ in the gut of one healthy infant-mother pair. Two fecal metagenomic libraries, one from the mother and one from her exclusively breast-fed infant one month after birth [9], were screened for fosmid clones conferring Tc^r^, which were further screened by PCR for a battery of Tc^r^ genes. End-sequencing established the microbial diversity among the Tc^r^ organisms. Finally we identified a novel Tn*916*-like conjugative transposon, Tn*6079* carrying Tc^r^ resistance genes *tet*(M) and *tet*(L) and the erythromycin resistance gene *erm*(T) in the infant gut.

## Results

### Screening metagenome libraries from infant and mother for clones conferring Tc^r^

The metagenome libraries from the infant and the mother contained 44 and 272 fosmid Tc^r^ clones respectively. In a first instance, we screened all obtained Tc^r^ clones for the common ribosomal protection genes *tet*(M), *tet*(O), *tet*(W) and *tet*(S). Out of the 44 Tc^r^ fosmid clones from the infant library, 43 were shown to be positive for *tet*(M) by PCR. One of the end-sequences (B04-U-PCC1R, 386 bp) from the fosmid clone negative for the *tet*(M) PCR was identical to a region in *tet*(M) downstream of one of the screening primers. Thus all 44 Tc^r^ clones from the infant's metagenomic library were *tet*(M) positive and negative for *tet*(S), *tet*(O) and *tet*(W). In contrast, out of the 272 Tc^r^ clones from the mother library, 21 (7.7%) were only positive for *tet*(W) and 204 (75%) were only positive for *tet*(O); for 47 (17.3%) of the Tc^r^ clones none of the assayed Tc^r^ genes were detected, and all clones were negative for *tet*(M) and *tet*(S).

To further investigate what resistance genes might be present in the 47 maternal clones that were negative for *tet*(M), *tet*(O), *tet*(W) and *tet*(S), we performed a series of multiplex PCRs designed to detect *tet*(A), *tet*(B), *tet*(C), *tet*(D), *tet*(E), *tet*(G), *tet*(K), *tet*(L), *tet*A(P), *tet*(Q) and *tet*(X). This second round of PCR screening detected *tet*(X), encoding a tetracycline-inactivating enzyme, in 17 of the tested clones and none of the other genes assayed. Overall, our PCR screens were able to account for the Tc^r^ genes present in 242 (89%) of the Tc^r^ clones from the mother's metagenomic library.

Sequencing of all the PCR screening products for *tet*(M) detected in the infant library identified a single sequence type, *tet*(M)a, based on 505 bp out of the 1920 bp of the *tet*(M) gene (Table S1). All 21 *tet*(W) PCR screening products, 63 of the 204 products for *tet*(O) and 12 of the 17 *tet*(X) products from the maternal library were also sequenced (Table S2). Of the sequenced *tet*(O) products, 13 were selected to represent genes assigned to different families/genera (see later in the Results section) and the remaining 50 were randomly selected. Based on 609 bp and 499 bp out of the 1920 bp of *tet*(W) and *tet*(O) and 446 bp out of the 1161–1167 bp of *tet*(X), 2 (*tet*(W)a, b), 9 (*tet*(O)a–i) and 1 (*tet*(X)a) different sequence types were identified (Table S2). The sequenced PCR screening products, *tet*(M)a and *tet*(O)a–i could discriminate among the known variants of *tet*(M) and *tet*(O) (Fig. S1, S2). *tet*(W)a and *tet*(W)b could discriminate between groups with highly related *tet*(W) genes sharing 99.9–100% and 99.5–100% sequence identity, respectively (Fig. S3). *tet*(X)a could discriminate among most known variants (Fig. S4) and was identical to the corresponding fragments from two *tet*(X) genes identified in *Bacteroides*, including the *tet*(X) gene first detected in transposon Tn*4351*/Tn*4400* [16].

### PCR screening of the infant's fecal DNA for the presence of *tet*(W), *tet*(O) and *tet*(X)

Although *tet*(W), *tet*(O) and *tet*(X) were not detected in the infant's metagenomic library, these genes could nonetheless have been present in the infant GIT microbiome, perhaps in non-abundant species that were not captured in the library. To further investigate the possibility of maternal transmission of resistances to the infant, we PCR-screened the total DNA from the infant fecal sample from which the metagenomic library was constructed. *tet*(X) was not detected, but, remarkably, we obtained amplifications in both the *tet*(W) and the *tet*(O) screening PCR's, albeit the *tet*(W) product could only be observed as a very faint band after a standard number of screening PCR cycles. Both products yielded clean sequence reads, indicating that single sequence types were present in the amplicons. These screening products (*tet*(W)_infant_plug and *tet*(O)_infant_plug) were shown to be identical to *tet*(W)a and *tet*(O)h detected in the maternal metagenomic library (Fig. S2 and S3). This strongly indicates mother to infant transmission of specific *tet*(W) and *tet*(O) genes.

### Identification of a novel composite Tn*916*/*1545*-like conjugative transposon carrying *tet*(M), *tet*(L) and *erm*(T) in the infant library

**Figure 1:**
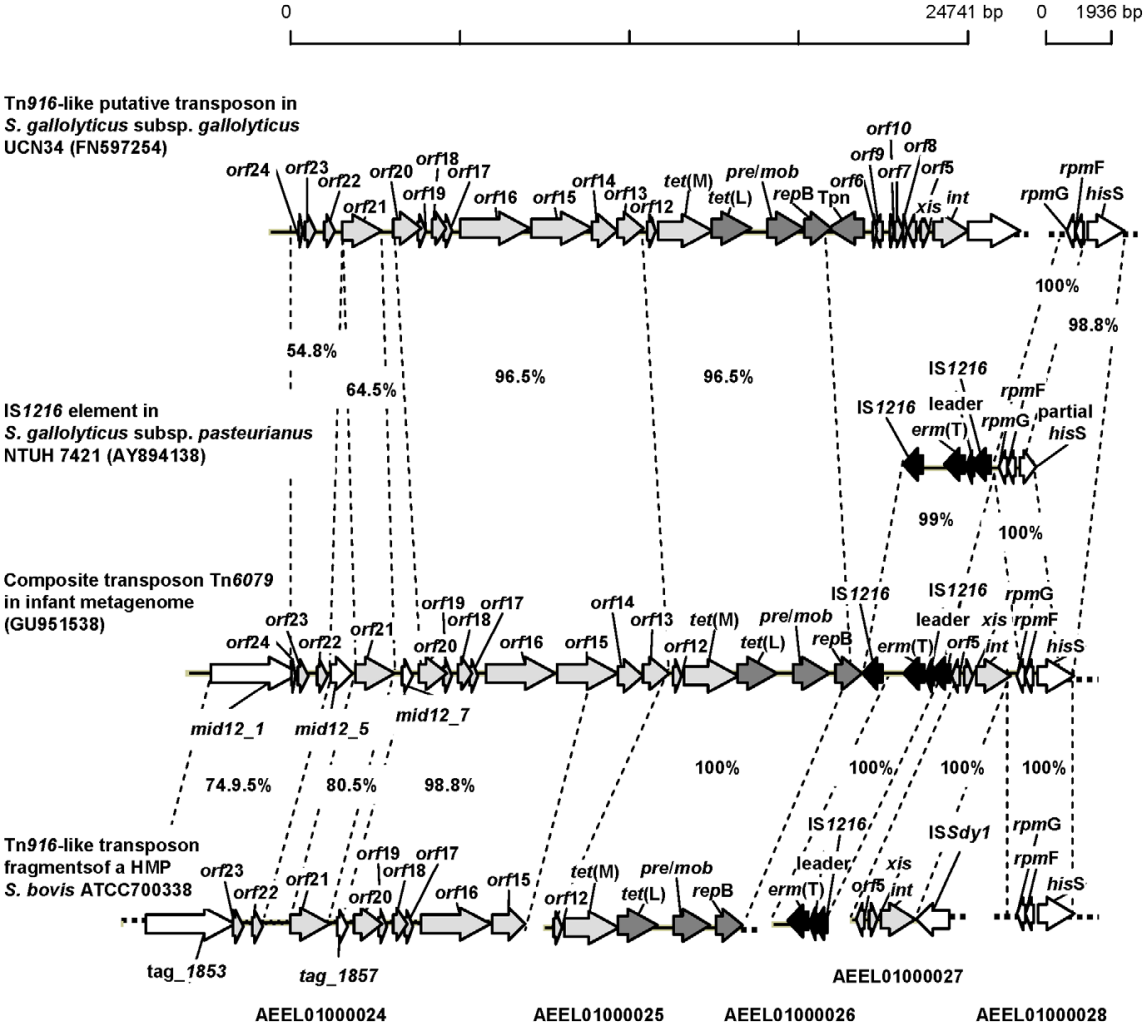
Composite transposon Tn*6079* from the infant metagenome compared to corresponding sequences from *S. gallolyticus* strains. Part of the sequenced fosmid-insert in the infant Tc^r^ metagenome (GU951538; 1–3095) is compared with the most similar homologous sequences in current databases, from *S. gallolyticus* subsp. *gallolyticus* strain UCN34 (FN597254; minus strand 564709–589025 and 52545–55152), *S. gallolyticus* subsp. *pasteurianus* NTUH7421 (AY894138; minus strand 1–4107) and *S. bovis* ATCC700338 (AEEL01000024; 112037–123526, AEEL01000025; 1–6541, AEEL01000026; 1–1578, AEEL01000027; 1–3192, AEEL01000028; 37166–39101). The relationships between sequences are shown as percentage identity at nucleotide level, calculated with the EMBOSS program Needle (http://www.ebi.ac.uk/Tools/emboss/align/index.html). Light gray arrows represent ORFs with homology to ORFs from Tn*916*/Tn*1545*-like conjugative transposons: *orf*5–10, *orf*12–24, *tet*(M), an excisionase (*xis*) and an integrase (*int*) of Tn*6079*. Dark gray arrows illustrate ORFs thay may be of plasmid origin: *tet*(L), *pre/mob* and *rep*. Black arrows illustrate ORFs that appear to be inserted by the two identical IS*1216*-like elements: first IS*1216*, *erm*(T), *erm*(T) leader sequence and second IS*1216*. *rmp*G and *rmp*F were predicted to encode L33 and L32 of the 50S ribosomal subunit. The functions of the predicted ORFs, *mid12_1*, *mid12_5* and *mid12_7* are unknown. *his*S: histidyl-tRNA synthetase. IS*Sdy1*: putative transposase.

In a sequenced fosmid from the infant library, *tet*(M) was found on a Tn*916/1545*-like transposon, a family of conjugative transposons that have an extremely broad host range [17], [18]. The transposon was highly similar to a putative Tn*916*-like transposon identified in *Streptococcus gallolyticus* subsp. *gallolyticus* strain UCN34 (FN597254), isolated from an elderly endocarditis and colon cancer patient [19], although the infant transposon was located at the 3′end of *rpm*G (predicted to encode protein L33 from the ribosomal 50S subunit), whereas the transposon from strain UCN34 was located in a putative peptidoglycan-linked protein (Fig. 1). Both transposons contained a second Tc^r^ gene, *tet*(L), predicted to encode an efflux protein, closely linked to plasmid recombination/mobilization (*pre/mob*) and replication (*rep*) genes. A DNA fragment containing the Tn*916-*like *orf*12 as well as *tet*(M), *tet*(L), *pre*/*mob* and most of *rep*B (see Fig. 1) has also recently been deposited in GenBank (AEEL01000025, contig of 6541 bp) as part of the draft sequence of a Human Microbiome Project (HMP) strain characterized as *S. bovis* ATCC 700338 and isolated from the vagina. This fragment is 100% identical to the homologous region in the infant transposon. Additionally, another DNA fragment from the same *S. bovis* draft sequence (AEEL01000027, contig of 35283 bp) contained a region (1900 bp) with Tn*916*-like *orf*5, *xis* and *int* that was 100% identical with a homologous region in the infant transposon (Fig. 1). This Tn*916*-like region was located at the 3′end of putative transposase IS*Sdy1*. The infant transposon also contained a 3026 bp sequence encoding an erythromycin rRNA methylase gene, *erm*(T), surrounded by two putative IS*1216* transposase genes, not present in strain UCN34. This 3026 bp sequence was 100% identical, except for an additional 30 bp between the *erm*(T) leader and the second IS*1216* element (overall DNA identity of 99%), to a corresponding fragment from *S. gallolyticus* subsp. *pasteurianus* NTUH 7421 (AY894138) [20] (Fig. 1). A fragment from *S. bovis* ATCC 700338 (AEEL01000026, contig of 1578 bp) containing *erm*(T), leader and an IS*1216* was 100% identical with the homologous region in the infant transposon (Fig. 1).

At the genic level, the *tet*(M) sequence in the infant library insert was also highly similar to previously identified *tet*(M) genes from the composite transposons, CTn*6002*, identified in *Streptococcus cristus* (AY898750, 99.9% DNA identity), and Tn*1545*, first identified in *Streptococcus pneumoniae* BM420 (AM889142, 98.2% DNA identity) [21], [22]. The infant *tet*(L) gene was shown to share 98.1% DNA identity (1.4% gaps) with *tet*(L) genes found on *pre/mob-* and *rep-*containing plasmids in *Bacillus stearothermophilus* (M63891), *Enterococcus faecalis* (AF503772) and *Staphylococcus aureus* (FM207105), as well as with chromosomally encoded *tet*(L) genes located just downstream of *tet*(M) and upstream of *pre/mob* in *Streptococcus suis* (FM252032) and *S. gallolyticus* subsp. *gallolyticus* strain UCN34 (Fig. 1).

Thus we have identified a novel composite Tn*916*/*1545*-like conjugative transposon which we registered as Tn*6079* in the Transposon Nomenclature Database from the UCL Eastman Dental Institute, London (http://www.ucl.ac.uk/eastman/tn/) [23].

### Tc^r^ was conferred exclusively by *tet*(M) and/or *tet*(L) from streptococci in the infant metagenomic library

**Figure 2:**
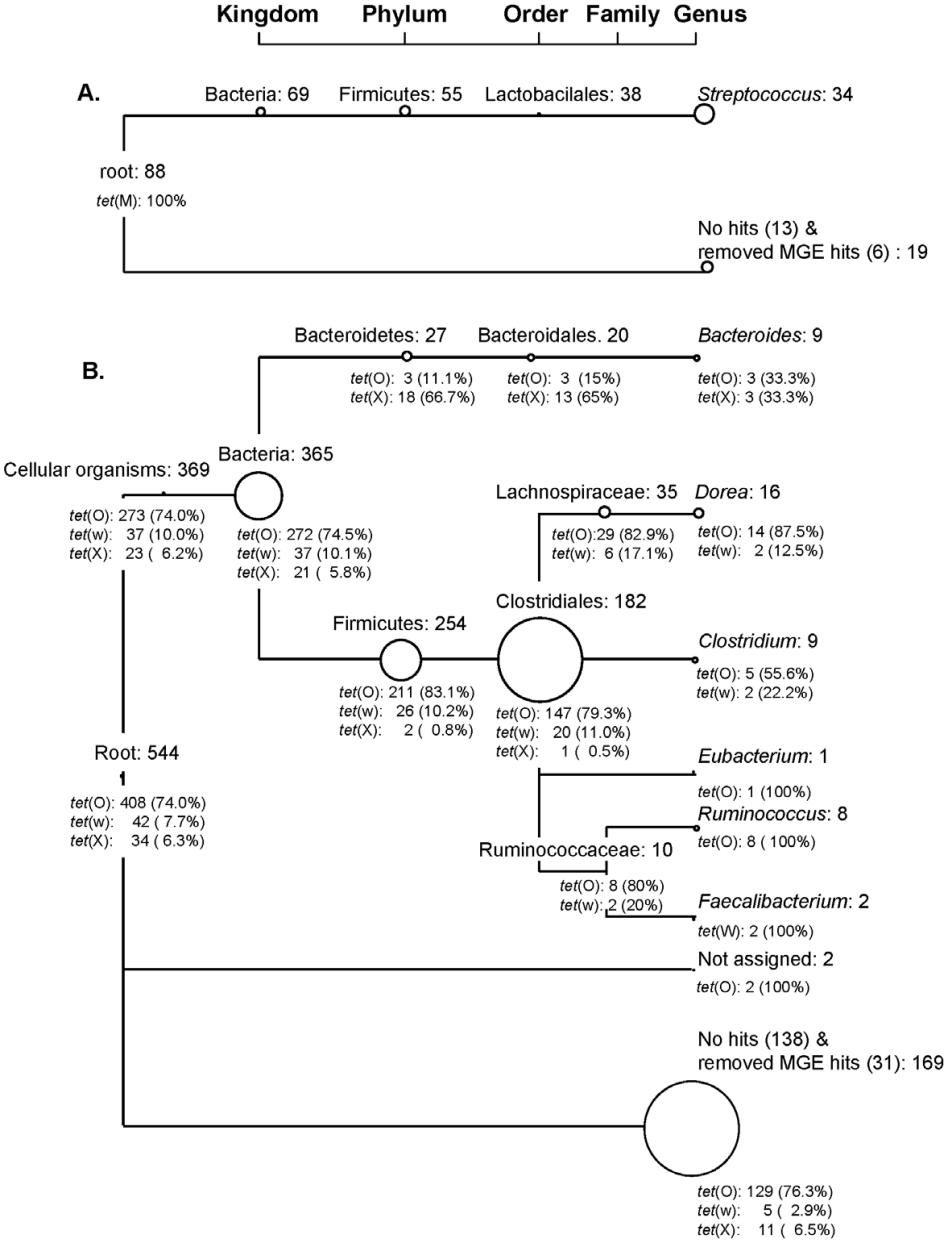
Microbial diversity of Tc^r^ fosmid clones in infant (A) and mother (B) metagenomes. Modified MEGAN tree (collapsed at Genus level) showing summarized number of reads assigned at different taxonomical levels. The size of each node is proportional to the number of reads assigned to the node. Beneath each node the number and percentage of Tc^r^ genes detected in this study are noted. **A.** The “No hits & removed MGE hits” category contains 13 reads with no BLASTX hits (or hits that did not attain the min score/length of 0.15), 2 removed reads which were predicted to be located in MGE and were initially assigned below order level and 4 reads that mapped to ORFs in the sequenced transposon (Tn*6079*). **B.** The “No hits & removed MGE hits” category contains 138 reads with no BLASTX hits (or hits that did not attain the min score/length of 0.15) and 31 removed reads which were predicted to be located in MGE and were initially assigned below order level. The “Not assigned” category contains 2 reads that were assigned by BLASTX hits to uncultured bacteria.

In the infant Tc^r^ metagenomic library, 97.7% (43/44) of the fosmids had at least one of their end-reads assigned within bacteria and at the genus level 72.7% (32/44) of the fosmids were assigned within *Streptococcus* (Fig. 2A and Table 1). This was further supported by a comparison showing 100% DNA identity between the *rpmG* and *rpmF* ORFs from the sequenced infant fosmid, predicted to encode the 50S ribosomal subunit proteins L33 and L32, and the corresponding regions from the three *Streptococcus* strains in Fig. 1 containing similar MGE fragments (*S. gallolyticus* subsp. *pasteurianus* strain NTUH 7421, *S. gallolyticus* subsp. *gallolyticus* strain UCN34 and *S. bovis* ATCC 700338).

**Table 1:** Assignment of end-reads and corresponding fosmids from the infant Tc^r^ metagenome at different taxonomical levels.

Level of assignment	Assignment details	No. of reads (percentage of total reads: 88)	No. of fosmids (percentage of total fosmids: 44)
Kingdom	Reads/fosmids of which at least one end-read was assigned within Bacteria.	69 (78.4%)	43 (97.7%)
Phylum	Reads/fosmids of which at least one end-read was assigned within Firmicutes.	55 (62.5%)	39 (88.6%)
Order	Reads/fosmids of which at least one end-read was assigned within Lactobacillales.	38 (43.2%)	36 (81.8%)
Family	Reads/fosmids of which at least one end-read was assigned within Streptococcaceae.	38 (43.2%)	36 (81.8%)
Genus	Reads/fosmids of which at least one end-read was assigned within *Streptococcus*.	34 (38.6%)	32 (72.7%)
No hit & removed MGE hits	Both forward and reverse fosmid end-reads in “No hit & removed MGE hits”	2 (2.3%)	1 (2.3%)
	Both forward and reverse fosmid end-reads with no blastx hits or hits below min cut off (0.15 bit score/length).	0	0
	Both forward and reverse fosmid end-reads with no blastx hits.	0	0

Out of the 44 infant fosmids, 43 were positive for a PCR designed to amplify the *rpmG* and *rpmF* ORFs region from the sequenced fosmid (Fig. 1). The reverse primer used in this PCR was specifically designed to target a sequence just downstream of the *rpmF* ORF that was present in the sequenced fosmid and in *S. gallolyticus* subsp. *pasteurianus* strain NTUH 7421 and S. bovis ATCC 7000338 (AEEL01000028) but not in *S. gallolyticus* subsp. *gallolyticus* strain UCN34. The reverse end-sequence from the only fosmid that was negative for this PCR (B04-M32-PCC1R, 625 bp) mapped to a region containing the Tn*916*-like *orf*5 in the sequenced transposon, which showed that this insert ended within this ORF (see Fig. 1). All 44 fosmids were positive for *tet*(L) and *erm*(T) PCR screenings. Alignment of sequences for 5 randomly selected PCR products containing *rpmG* and *rpmF* showed 100% identity (over 634 bp) with the sequenced fosmid (see Fig. 1). Thus the 44 Tc^r^ fosmids from the infant probably represent fragments from the same *S. gallolyticus*–like genomic region having different fragment-specific start and end points.

### Tc^r^ was conferred mainly by Firmicutes and Bacteroidetes in the mother metagenome

In the adult Tc^r^ metagenome, 83.1% (226/272) of the fosmids had at least one of their end-reads assigned within Bacteria (Fig. 2B and Table 2). For 9.9% (27/272) of the fosmids neither of the end-reads had a BLASTX hit even though the majority (17/27 = 62.9%) of these had a read length >500 bp. At the Phylum/Order level, fosmids were mainly assigned within Firmicutes/Clostridales (66.2%/51.1%) compared to a smaller group of fosmids assigned within Bacteroidetes/Bacteroidales (8.1%/7.0%). At the Family/Genus level fosmids were assigned to Lachnospiraceae/*Dorea* (10.7%/4.4%), *Clostridium* (3.3%), *Eubacterium* (0.4%), *Ruminococcus* (2.9%), *Faecalibacterium* (0.7%) and *Bacteroides* (3.3%) (Fig. 2B and Table 2).

**Table 2:** Assignment of end-reads and corresponding fosmids from the mother Tc^r^ metagenome at different taxonomical levels.

Level of assignment	Assignment details	No. of reads (percentage of total reads: 544)	No. of fosmids (percentage of total fosmids: 272)
Kingdom	Reads/fosmids of which at least one end-read was assigned within Bacteria.	365 (67.1%)	226 (83.1%)
Phylum	Reads/fosmids of which at least one end-read was assigned within Bacteroidetes.	27 (5.0%)	22 (8.1%)
	Reads/fosmids of which at least one end-read was assigned within Firmicutes.	254 (46.7%)	180 (66.2%)
Order	Reads/fosmids of which at least one end-read was assigned within Bacteroidales.	20 (3.7%)	19 (7.0%)
	Reads/fosmids of which at least one end-read was assigned within Clostridales.	182 (33.4%)	139 (51.1%)
Family	Reads/fosmids of which at least one end-read was assigned within Bacteroidaceae.	9 (1.7%)	9 (3.3%)
	Reads/fosmids of which at least one end-read was assigned within Lachnospiraceae.	35 (6.4%)	29 (10.7%)
	Reads/fosmids of which at least one end-read was assigned within Clostridiaceae.	9 (1.7%)	9 (3.3%)
	Reads/fosmids of which at least one end-read were assigned within Eubacteriaceae.	1 (0.2%)	1 (0.4%)
	Reads/fosmids of which at least one end-read was assigned within Ruminococcaceae.	10 (1.8%)	10 (3.7%)
Genus	Reads/fosmids of which at least one end-read was assigned within *Bacteroides*.	9 (1.7%)	9 (3.3%)
	Reads/fosmids of which at least one end-read was assigned within *Dorea*.	16 (2.9%)	12 (4.4%)
	Reads/fosmids of which at least one end-read was assigned within *Clostridium*.	9 (1.7%)	9 (3.3%)
	Reads/fosmids of which at least one end-read was assigned within *Eubacterium*.	1 (0.2%)	1 (0.4%)
	Reads/fosmids of which at least one end-read was assigned within *Ruminococcus*.	8 (1.5%)	8 (2.9%)
	Reads/fosmids of which at least one end-read was assigned within *Faecalibacterium*.	2 (0.4%)	2 (0.7%)
“No hit & removed MGE hits”	Both forward and reverse fosmid end-reads in “No hit & removed MGE hits”	92 (16.9%)	46 (16.9%)
	Both forward and reverse fosmid end-reads with no blastx hits or hits below min cut off (0.15 bit score/length).	76 (13.9%)	38 (13.9%)
	Both forward and reverse fosmid end-reads with no blastx hits.	54 (9.9%)	27 (9.9%)

In regards to the Tc^r^ genes assayed in the mother, fosmids carrying *tet*(O) and *tet*(X) were assigned both within Bacteroidales and Clostridiales or to the group with no BLASTX hits (in this group both end-reads had lengths >500 bp for 11/18 = 61.1% of fosmids positive for *tet*(O) and for 3/3 = 100% of fosmid positive for *tet*(X)). Fosmids carrying *tet*(W) were only assigned within Clostridiales except for one fosmid having both end-reads assigned to the group with no BLASTX hits (lengths of forward and reverse end-reads were 128 and 219 bp, respectively) (Fig. 2B). Remarkably, fosmids containing *tet*(W) sequences type (a) with 100% identity were assigned within different families of the Clostridiales (Lachnospiraceae, Ruminococcaceae and Clostridiaceae) (Table S2). In addition, fosmids harboring identical sequences of types *tet*(O)b, *tet*(O)c or *tet*(O)d were also assigned within different families of this order (*tet*(O)b and *tet*(O)d within Lachnospiraceae and Clostridiaceae, *tet*(O)c within Lachnospiraceae and Ruminococcaceae). And most remarkably, fosmids containing *tet*(O)h and *tet*(X)a were assigned within different phyla of bacteria (orders Bacteroidales and Clostridiales) (Table S2). This may suggest that specific *tet*(W) and *tet*(O) genes have been horizontally transferred among different members of the Clostridiales and that specific *tet*(O) and *tet*(X) genes have transferred between bacteria belonging to different phyla.

## Discussion

This is a case study based on a functional screen for Tc^r^ fosmid clones from two previously prepared metagenomic libraries representing the gut microbiota from an infant and his mother one month after childbirth [9]. In correspondence with the lower complexity in the infant gut microbiota compared to the mother, we detected much fewer Tc^r^ fosmid clones in the infant metagenome than in the mother metagenome. The Tc^r^ genes detected in the infant metagenomic library did not represent a subset of those found in the mother, but rather a completely distinct set, belonging to a different gene class and encoded by a different species. However, total fecal DNA from the infant sample was shown to also contain specific Tc^r^ genes that were present in the maternal library (*tet*(W)a and *tet*(O)h), suggesting that these may have been transmitted from mother to son. Given that this DNA was not cloned, we can not determine the organisms that carried these genes in the infant, but phylogenetic assignment of maternal fosmids suggests that they may have been present in organisms belonging to the Clostridiales (and/or to *Bacteroides* in the case of *tet*(O)h).

In the infant library, *tet*(M) and *tet*(L) were detected in all the Tc^r^ fosmids whereas mainly *tet*(O) but also *tet*(W) and *tet*(X) were detected among the Tc^r^ fosmids from the mother. Although the approach employed here can only detect Tc^r^ genes that can be expressed in the *E. coli* library host, the prevalence of Tc^r^ genes observed in this study is in general agreement with former culture-independent studies that analyzed Tc^r^ in Europe directly by PCR or microarray hybridization [14], [15]. These works detected the *tet*(M) genotype to be abundant in Finnish infant fecal samples [15] and *tet*(O) and *tet*(W) to be the most prevalent Tc^r^ genes in fecal samples from adults in six different European countries as detected by microarray analysis [14]. In contrast, a functional metagenomic screen of antibiotic resistances in the gut of two adult individuals carried out in the USA (Boston, MA) recovered numerous *tet*(W) sequences but did not identify any *tet*(O) or *tet*(X) genes [24]. It is important to note that the screening approaches in this latter work and in our own study both require that Tc^r^ can be expressed in the *E. coli* library host strains at a level sufficient to confer resistance in the presence of the antibiotic, although each employs a different cloning vector. Our fosmid-based study has the potential disadvantage that resistance genes located on smaller plasmids (<40 kb) may not be represented in the metagenomic libraries, but, on the other hand, the larger insert size increases the likelihood to clone complete resistance genes and enables the recovery of complex genetic elements. The different results obtained in the two USA studies could be due to the different cloning systems and/or reflect the antibiotic concentrations used in the functional screenings (10 µg/ml tetracycline in our study versus 20 µg/ml tetracycline, oxytetracycline or minocycline in Sommer *et al*). In addition, functional screenings performed at even lower tetracycline concentrations might reveal further Tc^r^ genes that are weakly expressed.

The previous culture-independent analyses that identified Tc^r^ in human fecal samples did not investigate the bacterial species in which such resistance was encoded [14], [15], [24]. In our fosmid-based study, end-sequencing of fosmid inserts allowed for taxonomic identification of the resistant organisms present in the GIT of the two individuals analyzed. All infant Tc^r^ clones appeared to represent the same *Streptococcus* genomic region containing *tet*(M), *tet*(L) and *erm*(T) within a novel composite Tn*916*-like transposon, Tn*6079*, located at the 3′end of *rpm*G. The nucleotide sequences of both *rpm*G and its 5′ neighbor *rpm*F were 100% identical to those of *S. gallolyticus* subsp. *pasteurianus* strain NTUH 7421, *S. gallolyticus* subsp. *gallolyticus* strain UCN34 and *S. bovis* ATCC 700338 (Fig. 1). It is important to note that the heterogenous group of strains traditionally designated *S. bovis* has recently been split by modern taxonomic techniques into the sister species *S. gallolyticus* and *S. infantarius* [25]. Indeed, the 16S rRNA sequence of *S. bovis* ATCC 700338 shows 99 to 100% identity with the *S. gallolyticus* subsp. *pasteurianus* and *S. gallolyticus* subsp. *gallolyticus* 16S rRNA sequences currently available in GenBank. Therefore, the presence of *rpm*G and *rpm*F next to the Tn*6079* transposon in the sequenced infant fosmid allows for identification of the Tc^r^-carrying organism in the infant GIT to species level. The similarities in sequence and structure between Tn*6079* and corresponding MGE sequences in the *S. gallolyticus* subsp. *pasteurianus*, *S. gallolyticus* subsp. *gallolyticus* and *S. bovis* ATCC 700338 strains (Fig. 1) strongly suggest that the infant's composite transposon arose through a process involving intraspecific genetic exchange.

Regarding the origin of the *S. gallolyticus*-like strain carrying the transposon in the infant, this organism was probably not transmitted from the maternal GIT, since no streptococci were detected in the mother's fecal samples, neither in the resistance screens performed here, nor in the previous random end sequencing of the library [9], even though this species is a normal inhabitant in the GIT of humans and animals and can be isolated in 5–16% of fecal samples from healthy adults [26]. Possible origins may include transmission from other maternal areas that are known to often harbor streptococci, such as the skin, the birth canal and the mouth, from breast milk, where streptococci have also recently been detected [27], or from other individuals handling the infant. The 100% identities recovered between the sequenced fosmid insert and the vaginal strain *S. bovis* ATCC 700338 (see Fig. 1) suggest that the infant may have acquired this strain or a closely related one during his passage through the birth canal.

In the maternal library, microorganisms conferring Tc^r^ consisted mainly of Firmicutes and Bacteroidetes, which commonly represent the two major Phyla of the human GIT [28], [29] and were also the most represented in the fosmid library of the mother according to random end reads [9]. For 9.9% of the maternal Tc^r^ fosmids, neither of the end-reads had any BLASTX hits against the NCBI non-redundant protein database in spite of being of substantial length (>500 bp). These fosmids likely carry Tc^r^ genes, mainly *tet*(O), from microorganisms for which no close relatives have yet been cultured. *tet*(O) was the main gene conferring resistance and was detected both within the Clostridiales (Firmicutes) and also the Bacteroidales (Bacteroidetes), where it had not been reported previously. Similarly, *tet*(X) was detected within the Clostridiales and the Bacteroidales and fosmids carrying identical *tet*(O) or *tet*(X) sequences were assigned within both phyla/orders. *tet*(W) was present only within Clostridiales, but also for this order fosmids carrying 100% identical *tet*(W) sequences were assigned within three different families (Table S2). Sequence identity is not expected between genes that have been diverging as orthologs since the phylogenetic split between such distantly related bacteria and can therefore be interpreted as evidence of recent horizontal transfers among these organisms [30]–[32]. *tet*(W) genes and flanking sequences in different isolates of GIT bacteria from diverse hosts have also been shown to share a high degree of similarity in previous analyses [33]. However, this is the first time that exact sequences of an antibiotic resistance gene are shown to occur in distantly related bacteria naturally coexisting in the gut of a single person at a particular point in time. Although their coexistence does not prove that the horizontal transfers occurred in the GIT of the infant's mother, alternative explanations would still necessitate recent transfers, in the environment or in the GITs of other individuals, followed by colocalization of the bacteria in this individual's GIT. These scenarios would imply a high frequency of these exact sequences in nature and/or a high likelihood of colocalization of the bacteria carrying them, and therefore seem less parsimonious than *in situ* transfer among bacteria coexisting closely in the dense microbiota of the adult GIT. In addition to fosmids containing *tet*(O), *tet*(W) and *tet*(X), there was also a fraction of Tc^r^ maternal fosmids in which none of the assayed genes were detected (11%, assigned within Firmicutes or Bacteroidetes), and where Tc^r^ must have been conferred by rare resistance genes.

This study showed strong indications of transmission of specific Tc^r^ genes (*tet*(W)a and *tet*(O)h) from the mother's GIT to that of the infant. However, the third Tc^r^ gene present in the maternal genomic library, *tet*(X), was not detected in the infant. *tet*(W)a and *tet*(O)h could be found in the infant's uncloned fecal DNA but not in the infant metagenomic library, suggesting that they were only present in low numbers. This is supported by the fact that PCR with *tet*(W) screening primers produced only a very faint band, and by the detection of a single *tet*(O) sequence type in the infant out of the 9 different types detected in the mother (Fig. S2). The scarcity of maternal Tc^r^ genes in the infant could be partially explained by the fact that approximately half of the detected Tc^r^ in the mother library was encoded by clostridia, and previous analyses of random end sequences from these libraries showed that clostridia were not abundant in the mother and that they were not transmitted to the infant [9]. On the other hand, those analyses, as well as comparisons of fosmid sequences and *Bacteroides*-specific 16S PCR libraries, have shown transmission to the infant of the two *Bacteroides* phylotypes present in the mother [9]. Given this established phylotype transmission, the fact that *Bacteroides* were amply represented in both the mother and infant libraries and the presence of *tet*(O)h and *tet*(X)a genes in *Bacteroides*-assigned maternal fosmids, the lack of *Bacteroides*-encoded Tc^r^ in the infant's library suggests that 1) *tet*(O)h and/or *tet*(X)a were present in only a small fraction of the maternal *Bacteroides* population and/or that 2) *tet*(O)h- and *tet*(X)a-encoding *Bacteroides* were selected against during the transmission process or in the infant gut. In fact, the first proposition is likely true, as according to previous random end sequencing analyses, *Bacteroides* represent nearly 48% of the maternal fosmid clones [9], and therefore hundreds of Tc^r^-encoding fosmids would be expected in a 69,000-clone library if *tet*(O)h and/or *tet*(X)a were present in every *Bacteroides* cell (based on a genome size of 6.5 Mb); in contrast, only 3 and 18 end-reads were assigned to Bacteroidetes among the mother clones containing *tet*(O)h and *tet*(X)a, respectively (Fig. 2B and Table S2).

In summary, for the first time we have characterized the microbial diversity of Tc^r^ bacteria in human gut samples, by analyzing GIT fosmid libraries from a mother and her infant. The maternal and infant libraries contained different resistant taxa encoding distinct sets of genes, but some of the specific Tc^r^ genes present in the mother could be recovered from uncloned infant fecal DNA. This indicates that transmission of Tc^r^ genes from the mother's GIT to the infant likely occurred, but that, due to the complexity of the GIT microbiota, species and genes present in low numbers were missed in the infant metagenomic library in spite of its large size (>70,000 clones). The likely role of the human gut as a privileged environment for HGT has been previously recognized [8], but here we present the first documented cases of identical resistance genes that could be directly linked to distantly related bacteria coexisting in the GIT of the same individual. The finding of a transposon in the infant carrying *tet*(M), *tet*(L) and *erm*(T), belonging to a family of broad host-range transposons, implied a strong potential for the joint transfer of tetracycline and erythromycin resistance within the infant's gut. These findings reinforce the notion that the human GIT is currently a relevant environment for the spread of antibiotic resistances, even in the case of young infants that solely ingest maternal milk. Further analyses involving more mother-infant pairs will be required in order to establish whether the trends observed in this case study describe the general relationship between mother and infant antibiotic resistomes.

## Materials and Methods

### Sample collection and ethics statement

The infant and mother metagenomic fosmid libraries analyzed in this study were prepared from fecal samples obtained one month after the infant's birth [9]. The infant was a healthy male, vaginally delivered at full term at the University Medical Center of the University of Arizona in Tucson (USA). He was exclusively breast-fed for 5 months. Samples were collected at the University of Arizona, with informed written consent from the infant's parents, using protocols approved by the institutional review boards of the Lawrence Berkeley National Laboratory and the University of Arizona.

### Metagenomic fosmid libraries and preparation of master plates with pooled clones

The infant and mother metagenomic fosmid libraries analyzed consisted of approximately 76000 and 69100 clones, respectively [9]. Fosmid inserts were approximately 40 kb, thus the infant and mother libraries represent roughly 3 Gb of DNA each. Clones from the infant and mother metagenomic fosmid libraries were pooled resulting in a reduction from 198 and 180 library (384 wells) plates to 14 and 12 (384 wells) master plates, respectively. Each master plate was constructed by pooling 15 library plates into one master plate using a Plate Mate Plus from Matrix. Each well in the master plates contained 30–40 µl LB (Millers) broth supplied with 7.5% Glycerol and 2 µl from each of the original library plates.

### Phenotypic screening of library master plates for Tc^r^

All master plates were screened for clones conferring Tc^r^ in growth plates (384 wells) containing 60 µl LB (Millers) broth with 10 µg/ml tetracycline per well. Growth or no growth was detected after overnight incubation at 37°C. When growth was detected, each of the 15 clones from the original fosmid libraries that could be responsible for the observed resistance phenotype was tested for Tc^r^ separately as described above.

### Genotypic PCR screenings and sequencing

All Tc^r^ clones (44 infant and 272 mother clones) from the two metagenomic libraries were screened for *tet*(M), *tet*(S), *tet*(O) and *tet*(W) by PCR using primer pairs TetM-1D/TetM-2, tetW-1/tetW-2, TetS-1/TetS-2 and TetO-1/TetO-2 (Table S3) [34]. To detect other possible resistance genes in the maternal Tc^r^ clones that were negative for these screening primers, we performed a series of multiplex PCRs designed to detect *tet*(A), *tet*(B), *tet*(C), *tet*(D), *tet*(E), *tet*(G), *tet*(K), *tet*(L), *tet*A(P), *tet*(Q) and *tet*(X), using previously reported primer combinations and protocols [35]. In addition, purified DNA from the infant fecal sample from which the infant fosmid library was constructed was screened for the Tc^r^ genes detected in the mother (*tet*(W), *tet*(O) and *tet*(X)).

After characterization of transposon Tn*6079*, the 44 infant clones were also screened for *tet*(L) [36], for a region linking *erm*(T) to an IS element (1010 bp) and for a region containing the *rpmG* and *rpmF* ORFs (664 bp) using primer pairs TetL-1/TetL-2, ermG-2/IS1216V3-1 and <REO/tRNA_S (Table S3). The latter PCR primer pair was designed to specifically target the region starting just downstream of the *int* gene within Tn*6079* and ending downstream of the *rpm*F gene.

PCR screening products from both uncloned fecal DNA from the infant (*tet*(W) and *tet*(O)) and from each metagenomic library (*tet*(M), *tet*(W), *tet*(O) and *tet*(X)) were sequenced with the PCR primers by Macrogen, Korea (http://www.macrogen.com/eng/sequencing/sequence_main.jsp) (see Tables S1 and S2). In addition, 5 randomly selected PCR screening products containing the *rpmG* and *rpmF* ORFs (B04-M4, B04-M8, B04-M13, B04-M16 and B04-M18) were also sequenced. ClustalX [37] was used to align sequences within the *tet* groups to determine different sequence types (Tables S1 & S2) and to align the *rpmG* and *rpmF* sequences. All together, 13 different Tc^r^ gene sequence types were deposited in GenBank (accession no. HN150556–HN150563, HR941095–HR941098 and JN104731). Neighbor Joining (NJ) trees based on the total gene sequence of selected Tc^r^ genes (57 *tet*(M), 18 *tet*(O), 24 *tet*(W) and 26 *tet*(X)) from GenBank and NJ trees based on the sequenced PCR screening products (505 bp, 499 bp, 609 bp and 446 bp, respectively) were constructed in ClustalX [37]. The trees were compared in order to show to what degree the sequenced PCR screening products were able to discriminate among the known variants of *tet*(M), *tet*(O), *tet*(W) and *tet*(X).

### Sequencing a fosmid-insert carrying *tet*(M)

One fosmid carrying *tet*(M) from the infant library (B04-M2) was sequenced as part of a mix of 12 fosmids pyrosequenced with Multiple Sequence Identifiers (MIDs) in a Roche GS FLX instrument in the Sequencing Technology group of the DOE Joint Genome Institute (JGI), CA, USA (http://www.jgi.doe.gov/). Reads belonging to the B04-M2 fosmid were sorted out and assembled using the Roche 454 Newbler software. Seven contigs were generated of which five (lengths 23744 bp, 19861 bp, 6382 bp, 1578 bp and 809 bp) were used to assemble the fosmid sequence. The remaining two contigs (lengths 958 bp and 507 bp) were highly similar or identical to the *E. coli* host genome and therefore were not incorporated into the assembly. Ten sequencing primers, M1b, M2, M2b, M3, M4, M5, M6, C340F, C01F and C01R were designed (Table S3) and Sanger reads, produced by Macrogen, Korea, were used to close the remaining five gaps. FosmidMAX™ DNA Purification Kit (EPICENTRE, USA) was used to prepare fosmid DNA template for the Sanger sequencing reactions. A finished 53499 bp circular fosmid containing a 45066 bp insert was assembled. The insert was annotated by NCBIs ORF finder, visualized by Vector NTI 10 (Invitrogen) and deposited in GenBank (accession no. GU951538).

### End-Sequencing

Inserts from all Tc^r^ fosmid clones from the infant (44) and adult (272) library were sequenced at both ends using pEpiFOS forward (PCC1F) or T7 promoter sequencing (T7) primers and the pEpiFOS reverse primer (PCC1R) (Table S3). End-sequencing was performed by the Sanger method using BigDye Terminators in ABI 3730 sequencers at the JGI. Out of 632 end-reads, 543 high quality (Phred≥Q20) sequences [38], [39], with a minimum length of 100 bp were retained after being trimmed by the program Trim at Greengenes (http://greengenes.lbl.gov) [40]. The remaining 89 end-reads were resequenced by Macrogen, Korea, and quality checking (≥Q20) and trimming were performed manually in Vector NTI. Vector contaminations were removed from 16 end-reads prior to Genome Survey Sequences (GSS) submission to GenBank. All together 632 end-reads with lengths ranging from 100 bp to 811 bp were deposited in Genbank (accession no. HN149924–HN150555). Read lengths ranged from 500 bp to 799 bp for 84.1% and 86.4% of infant and mother Tc^r^ end-reads, respectively.

### Assignment of end-sequences

All together 632 high quality sequences were used as queries to establish bacterial diversity through BLASTX searches against the NCBI non-redundant protein database (e-value<e^−15^). End-sequencing has recently been validated as a reliable method of determining diversity in a metagenomic sample, as random sequence reads from fosmid libraries of human fecal samples provide results very similar to those obtained based on the analysis of 16S sequences [41]. Each of the two BLASTX results (mother and infant) were separately parsed and visualized using MEGAN (version 3.7.4) software (Min Score = 35, Min Score/Length = 0.15, Top Percent = 20, Min Support = 1) [42]. Min Score/Length = 0.15 was chosen in order to account for the different read lengths. Because end-sequences located in MGE could easily bias the bacterial assignment by MEGAN, the BLASTX results were parsed for reads with hits containing the regular expressions *conjugative*, *transposon*, *tn916*, *integrase*, *recombinase*, *excisionase*, *mobilization* and *resistance*, and if such reads were assigned below order-level they were manually removed (Tables S4 & S5). Additionally, 4 infant end-reads (B04-M19-PCC1F, B04-M20-PCC1F, B04-M29- PCC1R, B04-M33-PCC1R) that were not found by the parsing of the BLASTX result but mapped to ORFs in the sequenced transposon were also removed. The assignment of reads by MEGAN based only on one BLASTX hit is very sensitive to misclassified sequences in GenBank. Therefore the taxonomical classification of BLASTX hit sequences used by MEGAN to assign reads at species level was reviewed (Tables S6 & S7). Finally, it was checked that assignments of forward and reverse end-reads from the same fosmid did not contradict each other.

## Supporting Information

Figure S1 **NJ tree based on 505 bp corresponding to the sequenced PCR screening products of** ***tet*** **(M).** The tree includes 57 *tet*(M) genes from GenBank and sequence type *tet*(M)a (bold) found among Tc^r^ clones in the infant metagenomic library. *tet*(M)a differs from the 57 *tet*(M) genes present in GenBank at the time of screening.
(TIF)

Figure S2 **NJ** ***tet*** **(O) trees showing that** ***tet*** **(O)a–i can discriminate among the known variants of** ***tet*** **(O).** **A.** Tree based on 499 bp corresponding to the sequenced PCR screening products of *tet*(O). *tet*(O)a–i represent the nine sequence types found among 63/204 *tet*(O) fosmids from the maternal metagenomic library and *tet*(O)_infant_plug represents the sequence type detected directly in uncloned DNA from the infant fecal sample. **B.** Tree based on the total *tet*(O) gene (1920 bp) of 18 GenBank sequences defined as *tet*(O) by sharing ≥80% identity at the amino acid level. However, NC_006134 is a mosaic combination of *tet*(O) and *tet*(M).
(TIF)

Figure S3 **NJ trees showing to what degree** ***tet*** **(W)a,b can discriminate among the known variants of** ***tet*** **(W).** **A.** Tree based on 609 bp corresponding to the sequenced PCR screening products of *tet*(W). *tet*(W)a,b represent the two sequence types found among 21 *tet*(W) fosmids from the maternal metagenomic library and *tet*(W)_infant_plug represents the sequence type detected directly in uncloned DNA from the infant fecal sample. (DQ525023 is not included in group *tet*(W)b because the *tet*(W) screening primers are not specific for this gene). **B.** Tree based on the total *tet*(W) gene (1920 bp) of 24 GenBank sequences defined as *tet*(W) by sharing ≥80% identity at the amino acid level. However, AY485122, AY485126, AY196920, AY196921, and DQ525023 are different mosaic combinations of *tet*(W), *tet*(O) and *tet*(32) and the *tet*(W) screening primers are not specific for these genes.
(TIF)

Figure S4 **NJ trees showing to what degree** ***tet*** **(X)a can discriminate among the known variants of** ***tet*** **(X).** **A.** Tree based on 447 bp corresponding to the sequenced PCR screening products of *tet*(X). *tet*(X)a represents the single sequence type found among 12 sequenced *tet*(X) PCR screening products from the maternal metagenomic library. **B.** Tree based on the total *tet*(X) gene (1167 bp) of 26 GenBank sequences of *tet*(X).
(TIF)

Table S1 **Sequence type of 43** ***tet*** **(M) genes detected in the infant metagenome.**
(DOCX)

Table S2 **Sequence types among 21** ***tet*** **(W) and 63 out of a total of 204** ***tet*** **(O) genes detected in the mother metagenome.**
(DOCX)

Table S3 **Primers used in this study.**
(DOCX)

Table S4 **End-reads (17) from the infant Tc^r^ metagenome for which BLASTX hits contained the regular expressions** ***conjugative*** **,** ***transposon*** **,** ***tn916*** **,** ***integrase*** **,** ***recombinase*** **,** ***excisionase*** **,** ***mobilization*** **and** ***resistance*** **and therefore were predicted to be located in MGE (2 end-reads in bold letters were assigned below order level and therefore removed from their initial assignments to the group with no hits in** **figure 2A** **).**
(DOCX)

Table S5 **End-reads (141) from the mother Tc^r^ metagenome for which BLASTX hits contained the regular expressions** ***conjugative*** **,** ***transposon*** **,** ***tn916*** **,** ***integrase*** **,** ***recombinase*** **,** ***excisionase*** **,** ***mobilization*** **and** ***resistance*** **and therefore were predicted to be located in MGE (31 end-reads in bold letters were assigned below order level and therefore removed from their initial assignments to the group with no hits in** **figure 2B** **).**
(DOCX)

Table S6 **Review of taxonomical classification of BLASTX hit sequences used by MEGAN to assign reads at species level in the infant Tc^r^ metagenome.**
(DOCX)

Table S7 **Review of taxonomical classification of BLASTX hit sequences used by MEGAN to assign reads at species level in the mother Tc^r^ metagenome.**
(DOCX)

## References

[1] Schumann A, Nutten S, Donnicola D, Comelli EM, Mansourian R (2005). Neonatal antibiotic treatment alters gastrointestinal tract developmental gene expression and intestinal barrier transcriptome.. Physiol Genomics.

[2] Ley RE, Turnbaugh PJ, Klein S, Gordon JI (2006). Microbial ecology: human gut microbes associated with obesity.. Nature.

[3] Manichanh C, Rigottier-Gois L, Bonnaud E, Gloux K, Pelletier E (2006). Reduced diversity of faecal microbiota in Crohn's disease revealed by a metagenomic approach.. Gut.

[4] O'Mahony C, Scully P, O'Mahony D, Murphy S, O'Brien F (2008). Commensal-induced regulatory T cells mediate protection against pathogen-stimulated NF-kappaB activation.. PLoS Pathog.

[5] Turnbaugh PJ, Hamady M, Yatsunenko T, Cantarel BL, Duncan A (2009). A core gut microbiome in obese and lean twins.. Nature.

[6] Salyers AA, Gupta A, Wang Y (2004). Human intestinal bacteria as reservoirs for antibiotic resistance genes.. Trends Microbiol.

[7] Favier CF, Vaughan EE, De Vos WM, Akkermans AD (2002). Molecular monitoring of succession of bacterial communities in human neonates.. Appl Environ Microbiol.

[8] Palmer C, Bik EM, DiGiulio DB, Relman DA, Brown PO (2007). Development of the human infant intestinal microbiota.. PLoS Biol.

[9] Vaishampayan PA, Kuehl JV, Froula JL, Morgan JL, Ochman H (2010). Comparative metagenomics and population dynamics of the gut microbiota in mother and infant.. Genome Biol Evol.

[10] Roberts MC (2005). Update on acquired tetracycline resistance genes.. FEMS Microbiol Lett.

[11] Chopra I, Roberts M (2001). Tetracycline antibiotics: mode of action, applications, molecular biology, and epidemiology of bacterial resistance.. Microbiol Mol Biol Rev.

[12] American Academy of Pediatrics (2003). Antimicrobial agents and related therapy..

[13] Roberts MC (2003). Acquired tetracycline and/or macrolide-lincosamides-streptogramin resistance in anaerobes.. Anaerobe.

[14] Seville LA, Patterson AJ, Scott KP, Mullany P, Quail MA (2009). Distribution of tetracycline and erythromycin resistance genes among human oral and fecal metagenomic DNA.. Microb Drug Resist.

[15] Gueimonde M, Salminen S, Isolauri E (2006). Presence of specific antibiotic (tet) resistance genes in infant faecal microbiota.. FEMS Immunol Med Microbiol.

[16] Speer BS, Salyers AA (1990). A tetracycline efflux gene on Bacteroides transposon Tn4400 does not contribute to tetracycline resistance.. J Bacteriol.

[17] Roberts AP, Mullany P (2009). A modular master on the move: the Tn916 family of mobile genetic elements.. Trends Microbiol.

[18] Rice LB (1998). Tn916 family conjugative transposons and dissemination of antimicrobial resistance determinants.. Antimicrob Agents Chemother.

[19] Rusniok C, Couve E, Da CV, El GR, Zidane N (2010). Genome sequence of Streptococcus gallolyticus: insights into its adaptation to the bovine rumen and its ability to cause endocarditis.. J Bacteriol.

[20] Tsai JC, Hsueh PR, Chen HJ, Tseng SP, Chen PY (2005). The erm(T) gene is flanked by IS1216V in inducible erythromycin-resistant Streptococcus gallolyticus subsp. pasteurianus.. Antimicrob Agents Chemother.

[21] Warburton PJ, Palmer RM, Munson MA, Wade WG (2007). Demonstration of in vivo transfer of doxycycline resistance mediated by a novel transposon.. J Antimicrob Chemother.

[22] Cochetti I, Tili E, Mingoia M, Varaldo PE, Montanari MP (2008). erm(B)-carrying elements in tetracycline-resistant pneumococci and correspondence between Tn1545 and Tn6003.. Antimicrob Agents Chemother.

[23] Roberts AP, Chandler M, Courvalin P, Guedon G, Mullany P (2008). Revised nomenclature for transposable genetic elements.. Plasmid.

[24] Sommer MO, Dantas G, Church GM (2009). Functional characterization of the antibiotic resistance reservoir in the human microflora.. Science.

[25] Beck M, Frodl R, Funke G (2008). Comprehensive study of strains previously designated Streptococcus bovis consecutively isolated from human blood cultures and emended description of Streptococcus gallolyticus and Streptococcus infantarius subsp. coli.. J Clin Microbiol.

[26] Alazmi W, Bustamante M, O'Loughlin C, Gonzalez J, Raskin JB (2006). The association of Streptococcus bovis bacteremia and gastrointestinal diseases: a retrospective analysis.. Dig Dis Sci.

[27] Collado MC, Delgado S, Maldonado A, Rodriguez JM (2009). Assessment of the bacterial diversity of breast milk of healthy women by quantitative real-time PCR.. Lett Appl Microbiol.

[28] Eckburg PB, Bik EM, Bernstein CN, Purdom E, Dethlefsen L (2005). Diversity of the human intestinal microbial flora.. Science.

[29] Turroni F, Ribbera A, Foroni E, van SD, Ventura M (2008). Human gut microbiota and bifidobacteria: from composition to functionality.. Antonie Van Leeuwenhoek.

[30] Barbosa TM, Scott KP, Flint HJ (1999). Evidence for recent intergeneric transfer of a new tetracycline resistance gene, tet(W), isolated from Butyrivibrio fibrisolvens, and the occurrence of tet(O) in ruminal bacteria.. Environ Microbiol.

[31] Scott KP, Melville CM, Barbosa TM, Flint HJ (2000). Occurrence of the new tetracycline resistance gene tet(W) in bacteria from the human gut.. Antimicrob Agents Chemother.

[32] Shoemaker NB, Vlamakis H, Hayes K, Salyers AA (2001). Evidence for extensive resistance gene transfer among Bacteroides spp. and among Bacteroides and other genera in the human colon.. Appl Environ Microbiol.

[33] Kazimierczak KA, Flint HJ, Scott KP (2006). Comparative analysis of sequences flanking tet(W) resistance genes in multiple species of gut bacteria.. Antimicrob Agents Chemother.

[34] Aarestrup FM, Agersø Y, Gerner-Smidt P, Madsen M, Jensen LB (2000). Comparison of antimicrobial resistance phenotypes and resistance genes in Enterococcus faecalis and Enterococcus faecium from humans in the community, broilers, and pigs in Denmark.. Diagn Microbiol Infect Dis.

[35] Ng LK, Martin I, Alfa M, Mulvey M (2001). Multiplex PCR for the detection of tetracycline resistant genes.. Mol Cell Probes.

[36] Aarestrup FM, Agersø Y, Ahrens P, Jørgensen JC, Madsen M (2000). Antimicrobial susceptibility and presence of resistance genes in staphylococci from poultry.. Vet Microbiol.

[37] Thompson JD, Gibson TJ, Plewniak F, Jeanmougin F, Higgins DG (1997). The CLUSTAL_X windows interface: flexible strategies for multiple sequence alignment aided by quality analysis tools.. Nucleic Acids Res.

[38] Ewing B, Hillier L, Wendl MC, Green P (1998). Base-calling of automated sequencer traces using phred. I. Accuracy assessment.. Genome Res.

[39] Ewing B, Green P (1998). Base-calling of automated sequencer traces using phred. II. Error probabilities.. Genome Res.

[40] DeSantis TZ, Hugenholtz P, Larsen N, Rojas M, Brodie EL (2006). Greengenes, a chimera-checked 16S rRNA gene database and workbench compatible with ARB.. Appl Environ Microbiol.

[41] Manichanh C, Chapple CE, Frangeul L, Gloux K, Guigo R (2008). A comparison of random sequence reads versus 16S rDNA sequences for estimating the biodiversity of a metagenomic library.. Nucleic Acids Res.

[42] Huson DH, Auch AF, Qi J, Schuster SC (2007). MEGAN analysis of metagenomic data.. Genome Res.

