# Supplementary figures and images for: The Gut as Reservoir of Antibiotic Resistance: Microbial Diversity of Tetracycline Resistance in Mother and Infant
Source: PLoS One. 2011 Jun 28;6(6):e21644. doi: 10.1371/journal.pone.0021644 (PMC3125294; doi:10.1371/journal.pone.0021644)

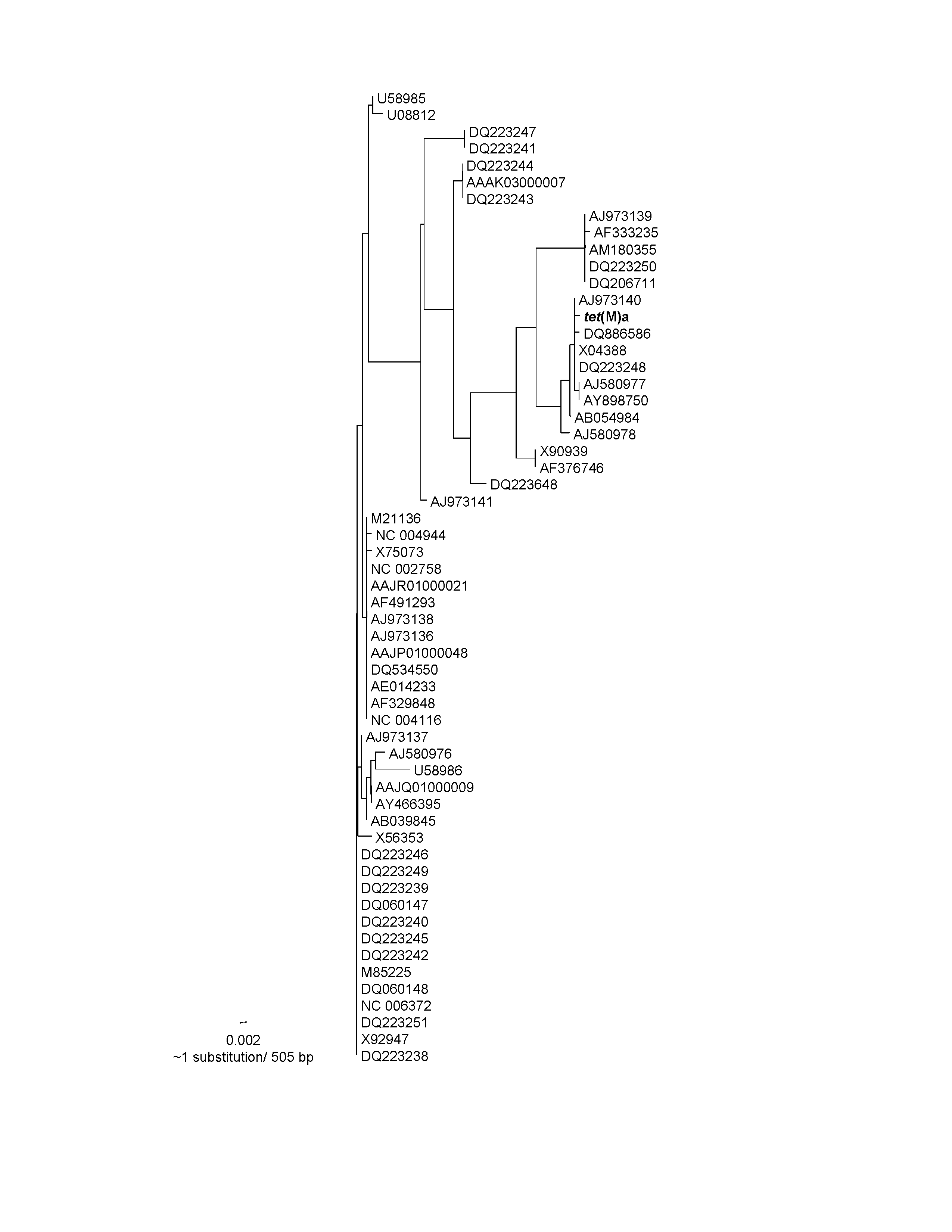

Supplement: Figure S1 — NJ tree based on 505 bp corresponding to the sequenced PCR screening products of tet (M). The tree includes 57 tet(M) genes from GenBank and sequence type tet(M)a (bold) found among Tcr clones in the infant metagenomic library. tet(M)a differs from the 57 tet(M) genes present in GenBank at the time of screening. (TIF) [file pone.0021644.s001.tif]

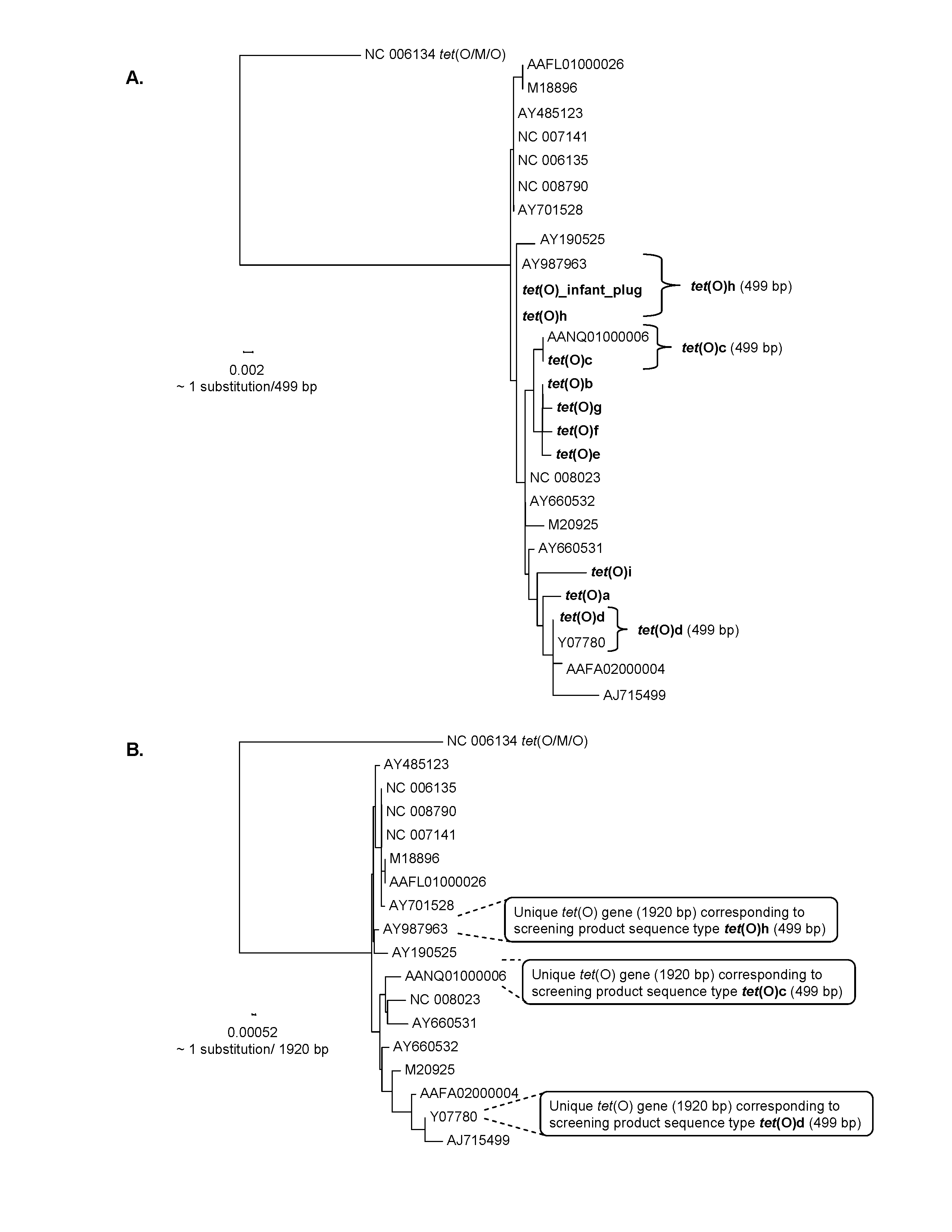

Supplement: Figure S2 — NJ tet (O) trees showing that tet (O)a–i can discriminate among the known variants of tet (O). A. Tree based on 499 bp corresponding to the sequenced PCR screening products of tet(O). tet(O)a–i represent the nine sequence types found among 63/204 tet(O) fosmids from the maternal metagenomic library and tet(O)_infant_plug represents the sequence type detected directly in uncloned DNA from the infant fecal sample. B. Tree based on the total tet(O) gene (1920 bp) of 18 GenBank sequences defined as tet(O) by sharing ≥80% identity at the amino acid level. However, NC_006134 is a mosaic combination of tet(O) and tet(M). (TIF) [file pone.0021644.s002.tif]

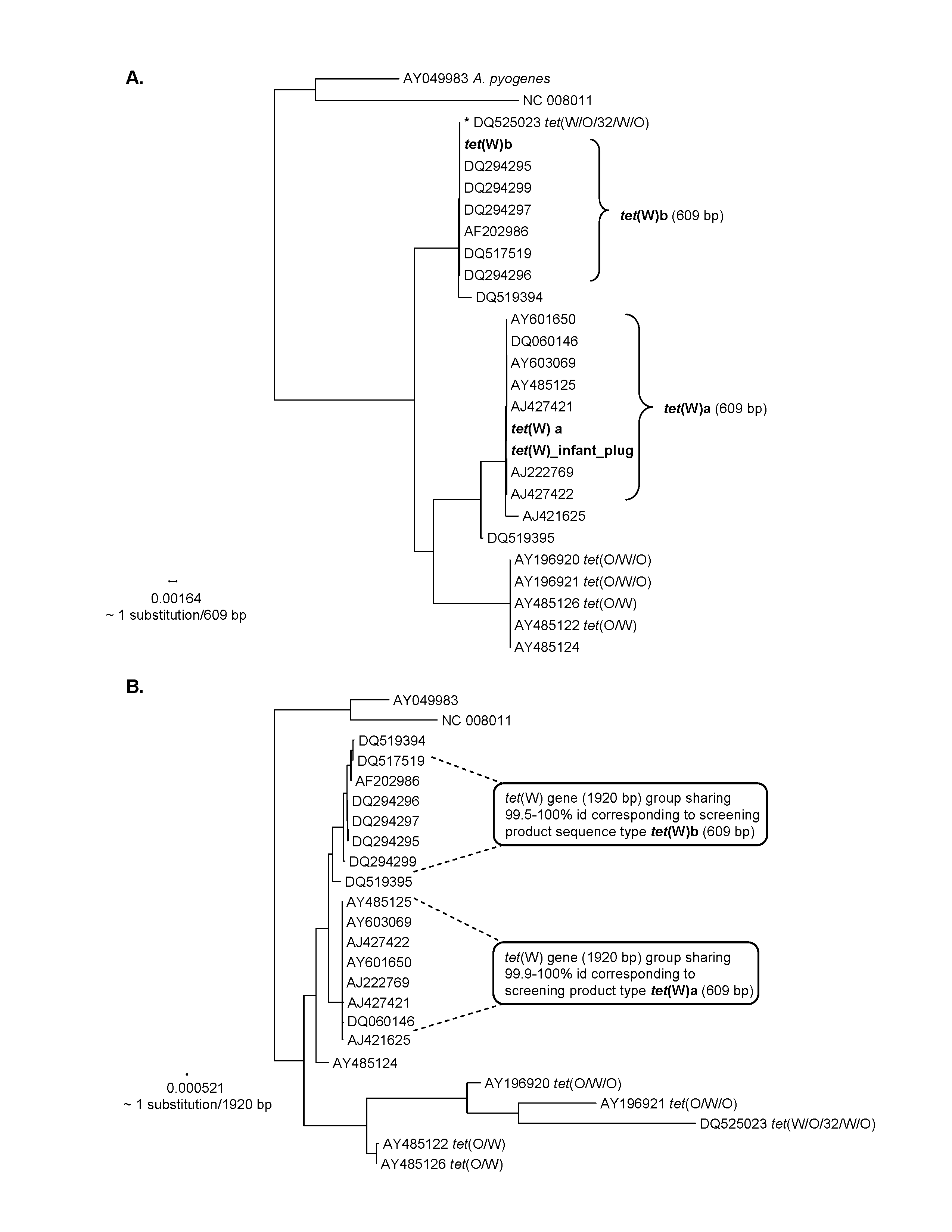

Supplement: Figure S3 — NJ trees showing to what degree tet (W)a,b can discriminate among the known variants of tet (W). A. Tree based on 609 bp corresponding to the sequenced PCR screening products of tet(W). tet(W)a,b represent the two sequence types found among 21 tet(W) fosmids from the maternal metagenomic library and tet(W)_infant_plug represents the sequence type detected directly in uncloned DNA from the infant fecal sample. (DQ525023 is not included in group tet(W)b because the tet(W) screening primers are not specific for this gene). B. Tree based on the total tet(W) gene (1920 bp) of 24 GenBank sequences defined as tet(W) by sharing ≥80% identity at the amino acid level. However, AY485122, AY485126, AY196920, AY196921, and DQ525023 are different mosaic combinations of tet(W), tet(O) and tet(32) and the tet(W) screening primers are not specific for these genes. (TIF) [file pone.0021644.s003.tif]

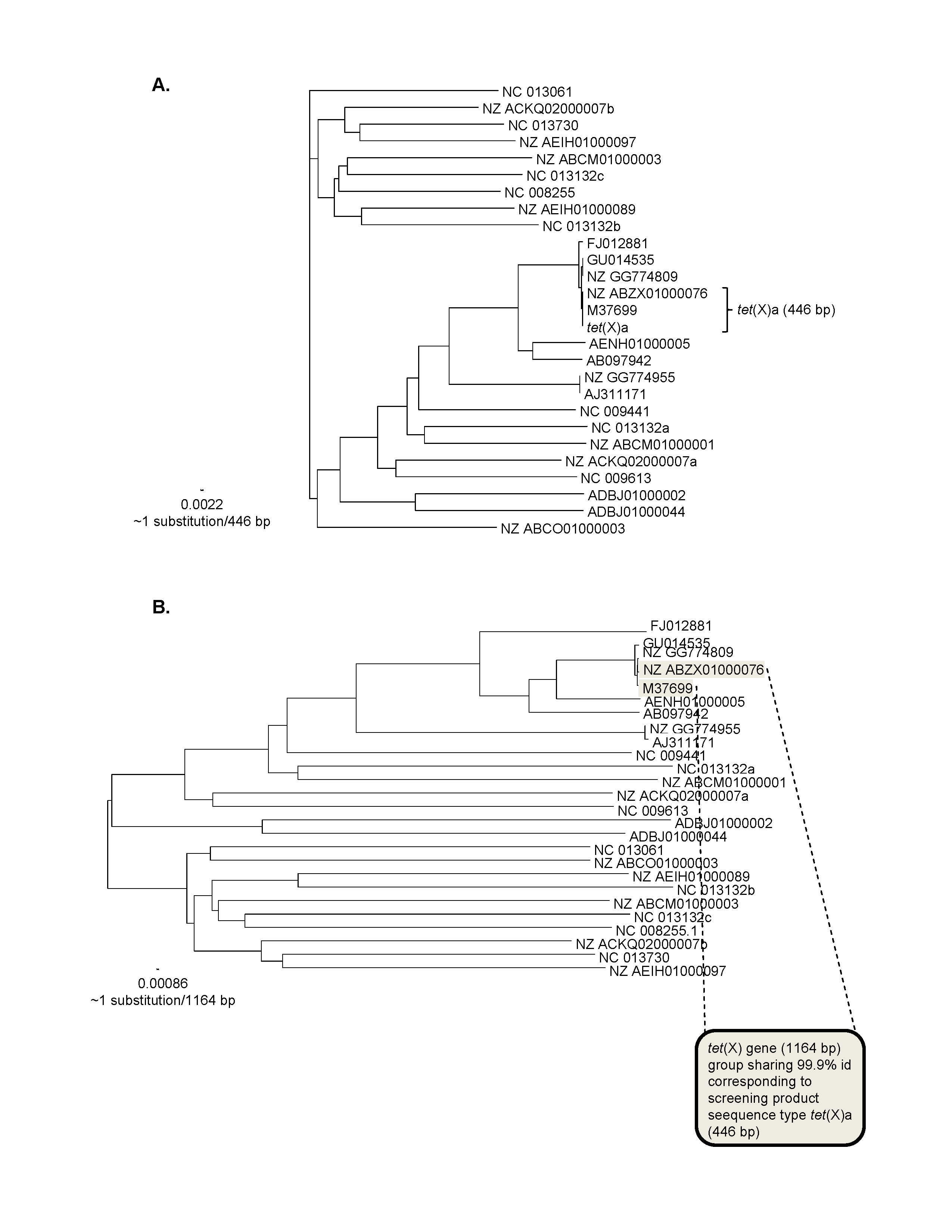

Supplement: Figure S4 — NJ trees showing to what degree tet (X)a can discriminate among the known variants of tet (X). A. Tree based on 447 bp corresponding to the sequenced PCR screening products of tet(X). tet(X)a represents the single sequence type found among 12 sequenced tet(X) PCR screening products from the maternal metagenomic library. B. Tree based on the total tet(X) gene (1167 bp) of 26 GenBank sequences of tet(X). (TIF) [file pone.0021644.s004.tif]
