# Supplementary material for: The Gut as Reservoir of Antibiotic Resistance: Microbial Diversity of Tetracycline Resistance in Mother and Infant
Source: PLoS One. 2011 Jun 28;6(6):e21644. doi: 10.1371/journal.pone.0021644 (PMC3125294; doi:10.1371/journal.pone.0021644)
Supplement: Table S1 — Sequence type of 43 tet (M) genes detected in the infant metagenome. (DOCX) [file pone.0021644.s005.docx]

**Table S1.** Sequence type of 43 *tet*(M) genes detected in the infant metagenome

| Fosmids with sequenced PCR screening product | Length of sequenced PCR screening products in bp | Sequence type | Number of fosmids with sequence type | Genbank accession no. |
| --- | --- | --- | --- | --- |
| B04-M1- B04-M43 | 505 | *tet*(M)a | 43 | HN150563 |
