# Supplementary material for: The Gut as Reservoir of Antibiotic Resistance: Microbial Diversity of Tetracycline Resistance in Mother and Infant
Source: PLoS One. 2011 Jun 28;6(6):e21644. doi: 10.1371/journal.pone.0021644 (PMC3125294; doi:10.1371/journal.pone.0021644)
Supplement: Table S2 — Sequence types among 21 tet (W) and 63 out of a total of 204 tet (O) genes detected in the mother metagenome. (DOCX) [file pone.0021644.s006.docx]

**Table S2.** Sequence types among 21 *tet*(W) and 63 out of a total of 204 *tet*(O) genes detected in the mother metagenome

| Fosmids with sequenced PCR screening product | Final assignment of forward/reverse end-reads | Length of sequenced PCR screening products in bp | Sequence type | Number of fosmids with sequence type | Genbank accession no. |
| --- | --- | --- | --- | --- | --- |
| tetW_W2 | Bacteria/Clostridiales | 609 | *tet*(W)a | 17 | HN150556 |
| tetW_W3 | Bacteria/Bacteria |  |  |  |  |
| tetW_W4 | Removed MGE hits/ Lachnospiraceae |  |  |  |  |
| tetW_W5 | *Dorea/*Lachnospiraceae |  |  |  |  |
| tetW_W6 | Bacteria/Firmicutes |  |  |  |  |
| tetW_W7 | Clostridiales/Bacteria |  |  |  |  |
| tetW_W9 | Clostridiales/*Faecalibacterium* |  |  |  |  |
| tetW_W10 | No hits/*Faecalibacterium* |  |  |  |  |
| tetW_W11 | Clostridiales/Firmicutes |  |  |  |  |
| tetW_W12 | *Clostridium*/No hits |  |  |  |  |
| tetW_W13 | Clostridiales/*Clostridium* |  |  |  |  |
| tetW_W14 | Firmicutes/Bacteria |  |  |  |  |
| tetW_W15 | Clostridiales/Clostridiales |  |  |  |  |
| tetW_W17 | Clostridiales/Bacteria |  |  |  |  |
| tetW_W18 | Clostridiales/Clostridiales |  |  |  |  |
| tetW_W19 | No hits/No hits |  |  |  |  |
| tetW_W20 | Lachnospiraceae/Lachnospiraceae |  |  |  |  |
| tetW_W1 | Bacteria/Firmicutes | 609 | *tet*(W)b | 4 | HN150557 |
| tetW_W8 | Bacteria/Firmicutes |  |  |  |  |
| tetW_W16 | Firmicutes/Bacteria |  |  |  |  |
| tetW_W21 | *Dorea*/Bacteria |  |  |  |  |
| tetO_452 | Firmicutes/Firmicutes | 499 | *tet*(O)a | 4 | HN150558 |
| tetO_482 | Clostridiales/No hits |  |  |  |  |
| tetO_532 | Bacteria/*Eubacterium* |  |  |  |  |
| tetO_727 | Clostridiales/Firmicutes |  |  |  |  |
| tetO_396 | No hits/No hits | 499 | *tet*(O)b | 24 | HN150559 |
| tetO_403 | Lachnospiraceae/Bacteria |  |  |  |  |
| tetO_411 | Clostridiales/Clostridiales |  |  |  |  |
| tetO_413 | No hits/No hits |  |  |  |  |
| tetO_420 | No hits/No hits |  |  |  |  |
| tetO_421 | Clostridiales/Clostridiales |  |  |  |  |
| tetO_430 | Bacteria/ No hits |  |  |  |  |
| tetO_432 | No hits/ Bacteria |  |  |  |  |
| tetO_435 | Clostridiales/Removed MGE hits |  |  |  |  |
| tetO_436 | Removed MGE hits/Clostridiales |  |  |  |  |
| tetO_459 | Clostridiales/Clostridiales |  |  |  |  |
| tetO_472 | Lachnospiraceae/Bacteria |  |  |  |  |
| tetO_483 | No hits/*Clostridium* |  |  |  |  |
| tetO_492 | Clostridiales/Bacteria |  |  |  |  |
| tetO_500 | No hits/No hits |  |  |  |  |
| tetO_539 | Clostridiales/Firmicutes |  |  |  |  |
| tetO_540 | No hits/No hits |  |  |  |  |
| tetO_556 | Clostridiales/Bacteria |  |  |  |  |
| tetO_596 | No hits/No hits |  |  |  |  |
| tetO_628 | Bacteria/No hits |  |  |  |  |
| tetO_639 | No hits/No hits |  |  |  |  |
| tetO_675 | No hits/No hits |  |  |  |  |
| tetO_701 | [Clostridiales](http://rdp.cme.msu.edu/classifier/hierarchy.jsp?root=211&depth=0&confidence=0.8)/No hits |  |  |  |  |
| tetO_754 | No hits/No hits |  |  |  |  |
| tetO_389 | Clostridiales/Lachnospiraceae | 499 | *tet*(O)c | 16 | HN150560 |
| tetO_392 | Firmicutes/Firmicutes |  |  |  |  |
| tetO_398 | Removed MGE hits/ *Ruminococcus* |  |  |  |  |
| tetO_405 | Removed MGE hits/ Firmicutes |  |  |  |  |
| tetO_407 | Firmicutes/ No hits |  |  |  |  |
| tetO_416 | No hits/ *Ruminococcus* |  |  |  |  |
| tetO_419 | Firmicutes/Firmicutes |  |  |  |  |
| tetO_464 | Removed MGE hits/ Clostridiales |  |  |  |  |
| tetO_467 | Clostridiales/Bacteria |  |  |  |  |
| tetO_470 | Bacteria/ No hits |  |  |  |  |
| tetO_485 | Lachnospiraceae/ Clostridiales |  |  |  |  |
| tetO_486 | [Clostridiales](http://rdp.cme.msu.edu/classifier/hierarchy.jsp?root=211&depth=0&confidence=0.8)/Removed MGE hits |  |  |  |  |
| tetO_487 | Firmicutes/[Clostridiales](http://rdp.cme.msu.edu/classifier/hierarchy.jsp?root=211&depth=0&confidence=0.8) |  |  |  |  |
| tetO_490 | Firmicutes/[Clostridiales](http://rdp.cme.msu.edu/classifier/hierarchy.jsp?root=211&depth=0&confidence=0.8) |  |  |  |  |
| tetO_494 | [Clostridiales](http://rdp.cme.msu.edu/classifier/hierarchy.jsp?root=211&depth=0&confidence=0.8)/ Removed MGE hits |  |  |  |  |
| tetO_747 | Not assigned/Firmicutes |  |  |  |  |
| tetO_438 | Firmicutes/Lachnospiraceae | 499 | *tet*(O)d | 5 | HN150561 |
| tetO_462 | *Dorea*/*Dorea* |  |  |  |  |
| tetO_480 | No hits/[Clostridiales](http://rdp.cme.msu.edu/classifier/hierarchy.jsp?root=211&depth=0&confidence=0.8) |  |  |  |  |
| tetO_677 | *Dorea*/Clostridiales |  |  |  |  |
| tetO_684 | *Clostridium*/Bacteria |  |  |  |  |
| tetO_427 | Clostridiales/No hits | 499 | *tet*(O)e | 1 | HN150562 |
| tetO_391 | No hits/No hits | 499 | *tet*(O)f | 1 | HR941095 |
| tetO_491 | Clostridiales/Bacteria | 499 | *tet*(O)g | 1 | HR941096 |
| tetO_431 | No hits/No hits | 499 | *tet*(O)h | 7 | HR941097 |
| tetO_439 | No hits/No hits |  |  |  |  |
| tetO_444 | Firmicutes/Bacteria |  |  |  |  |
| tetO_447 | Bacteroides/No hits |  |  |  |  |
| tetO_493 | Bacteria/Clostridiales |  |  |  |  |
| tetO_531 | Bacteroides/Bacteria |  |  |  |  |
| tetO_715 | No hits/Bacteroides |  |  |  |  |
| tetO_395 | [Clostridiales](http://rdp.cme.msu.edu/classifier/hierarchy.jsp?root=211&depth=0&confidence=0.8)/Removed MGE hits | 499 | *tet*(O)i | 4 | HR941098 |
| tetO_397 | Bacteria/Clostridiales |  |  |  |  |
| tetO_406 | Clostridiales/Clostridiales |  |  |  |  |
| tetO_445 | Bacteria/Cellular organisms |  |  |  |  |
| tetR_412 | No hits/No hits | 446 | *tet*(X)a | 12 | XXXXXXXX |
| tetR_446 | Bacteroides/No_hits |  |  |  |  |
| tetR_456 | Bacteroidetes/Bacteroides |  |  |  |  |
| tetR_460 | Clostridiales/Firmicutes |  |  |  |  |
| tetR_463 | Bacteroidales/Bacteroidales |  |  |  |  |
| tetR_541 | Bacteroidales/No hits |  |  |  |  |
| tetR_551 | No hits/Bacteroidetes |  |  |  |  |
| tetR_612 | No hits/No hits |  |  |  |  |
| tetR_659 | Bacteroidales/Bacteroidales |  |  |  |  |
| tetR_679 | No hits/Bacteroidetes |  |  |  |  |
| tetR_706 | Bacteroidales/No hits |  |  |  |  |
| tetR_731 | Bacteroidales/Bacteria |  |  |  |  |
