# Supplementary material for: The Gut as Reservoir of Antibiotic Resistance: Microbial Diversity of Tetracycline Resistance in Mother and Infant
Source: PLoS One. 2011 Jun 28;6(6):e21644. doi: 10.1371/journal.pone.0021644 (PMC3125294; doi:10.1371/journal.pone.0021644)
Supplement: Table S3 — Primers used in this study. (DOCX) [file pone.0021644.s007.docx]

**Table S3.** Primers used in this study

| Primer name | Sequence (5'- 3') |
| --- | --- |
| TetM-1 | GTTAAATAGTGTTCTTGGAG |
| TetM-1D^1^ | GYTAAATAGTGTTCKTGGAG |
| TetM-2^1^ | CTAAGATATGGCTCTAACAA |
| tetW-1^2^ | GCCATCTTGGTGATCTCC |
| tetW-2^2^ | TGGTCCCCTAATACATCGTT |
| TetS-1^1^ | TGGAACGCCAGAGAGGTATT |
| TetS-2^1^ | ACATAGACAAGCCGTTGACC |
| TetO-1^1^ | GATGGCATACAGGCACAGAC |
| TetO-2^1^ | CAATATCACCAGAGCAGGCT |
| TetL-1 | GTTGCGCGCTATATTCCAAA |
| TetL-2 | TTAAGCAAACTCATTCCAGC |
| <REO | CGAAAGCACATAGAATAAGGCTTTACGAGC |
| tRNA_S | ACGAACAGCCAAGCGTTAATAACCAC |
| ermG-2 (37) | TCTTTGTTAACCCATTTCAT |
| IS1216V3-1 | CCGAAGGTTGTAGTCACAGA |
| M1b | TCGGGAAGCGTGGAATG |
| M2r | AGGTTAATCTCCTATGGTGGT |
| M2b | ATCCTGTAAAGGATACGGATTTTATG |
| M3f | TCCGTCCTCGTTGTACCT |
| M4r | GCTTTGTATACCTATGGTTATGCA |
| M5f | ACCCTGATGCGACCAC |
| M6r | AGGCATTGATGGAATCGT |
| C340F | AGCACGCTTCCACGAAAGGA |
| C01F | TGACGATTTGCTGCCCTACCT |
| C01R | TAACCCACTCCTATGAGGT |
| PCC1F | GGATGTGCTGCAAGGCGATTAAGTTGG |
| T7 | TAATACGACTCACTATAGGG |
| PCC1R | CTCGTATGTTGTGTGGAATTGTGAGC |
| MID12_orf24 | GCTTCGGCGGAGAGCGT |

^1^ The screening primers for *tet*(M), *tet*(O) and *tet*(S) covered all naturally occurring determinants of there respective genes, found in GenBank at the time of screening (57 *tet*(M), 17 *tet*(O), 6 *tet*(S)). Screening primer, TetO-2 is not specific for the mosaic gene, *tet*(O/32/O) (NC_006134).

^2^ The *tet*(W) screening primers covered most of the naturally occurring tetW determinants found in GenBank (17/19 *tet*(W)), however primer *tet*W-2 had one mismatch for approximately half of these genes (8/17 *tet*(W)). The *tet*(W) screening primers are not specific for *tet*(W) from *Arcanobacterium pyogenes* (AY049983) and *tet*(W) from *Lawsonia intracellularis* (NC_008011) and for mosaic combinations of *tet*(O) and *tet*(W) such as *tet*(O/W) (AY485122 & AY485126), *tet*(O/W/O) (AY196920 & AY196921) and *tet*(W/O/32/WO) (DQ525023).
