# Supplementary material for: The Gut as Reservoir of Antibiotic Resistance: Microbial Diversity of Tetracycline Resistance in Mother and Infant
Source: PLoS One. 2011 Jun 28;6(6):e21644. doi: 10.1371/journal.pone.0021644 (PMC3125294; doi:10.1371/journal.pone.0021644)
Supplement: Table S4 — End-reads (17) from the infant Tcr metagenome for which BLASTX hits contained the regular expressions conjugative , transposon , tn916 , integrase , recombinase , excisionase , mobilization and resistance and therefore were predicted to be located in MGE (2 end-reads in bold letters were assigned below order level and therefore removed from their initial assignments to the group with no hits in figure 2A ). (DOCX) [file pone.0021644.s008.docx]

**Table S4.** End-reads (17) from the infant Tc^r^ metagenome for which BLASTX hits contained the regular expressions *conjugative*, *transposon*, *tn916*, *integrase*, *recombinase*, *excisionase*, *mobilization* and *resistance* and therefore were predicted to be located in MGE (2 end-reads in bold letters were assigned below order level and therefore removed from their initial assignments to the group with no hits in figure 2A).

| Query end-read |
| --- |
| Query= B04-M10-PCC1F |
| Query= B04-M12-PCC1F |
| Query= B04-M13-PCC1F |
| Query= B04-M16-PCC1F |
| Query= B04-M17-PCC1F |
| Query= B04-M18-PCC1F |
| Query= B04-M21-PCC1F |
| Query= B04-M27-PCC1F |
| Query= B04-M30-PCC1F |
| **Query= B04-M31-PCC1F** |
| **Query= B04-M32-PCC1R** |
| Query= B04-M34-PCC1R |
| Query= B04-M35-PCC1R |
| Query= B04-M36-PCC1R |
| Query= B04-M41-PCC1F |
| Query= B04-U-PCC1F |
| Query= B04-U-PCC1R |
