# Supplementary material for: The Gut as Reservoir of Antibiotic Resistance: Microbial Diversity of Tetracycline Resistance in Mother and Infant
Source: PLoS One. 2011 Jun 28;6(6):e21644. doi: 10.1371/journal.pone.0021644 (PMC3125294; doi:10.1371/journal.pone.0021644)
Supplement: Table S5 — End-reads (141) from the mother Tcr metagenome for which BLASTX hits contained the regular expressions conjugative , transposon , tn916 , integrase , recombinase , excisionase , mobilization and resistance and therefore were predicted to be located in MGE (31 end-reads in bold letters were assigned below order level and therefore removed from their initial assignments to the group with no hits in figure 2B ). (DOCX) [file pone.0021644.s009.docx]

**Table S5.** End-reads (141) from the mother Tc^r^ metagenome for which BLASTX hits contained the regular expressions *conjugative*, *transposon*, *tn916*, *integrase*, *recombinase*, *excisionase*, *mobilization* and *resistance* and therefore were predicted to be located in MGE (31 end-reads in bold letters were assigned below order level and therefore removed from their initial assignments to the group with no hits in figure 2B).

| Query end-read |
| --- |
| Query= tetW_FWF674.PCC1F.W1 |
| Query= tetW_FWF674.PCC1R.W1 |
| Query= tetW_FWF682.PCC1F.W2 |
| Query= tetW_FWF682.PCC1R.W2 |
| Query= tetW_FWF506.PCC1F.W3 |
| **Query= tetW_17_T7pro.W4** |
| Query= tetW_FWF755.PCC1R.w7 |
| Query= tetW_FWF388..PCC1F.w8 |
| Query= tetW_FWF471.PCC1F.W14 |
| Query= tetW_FWF592.PCC1F.W16 |
| Query= tetW_FWF592.PCC1R.W16 |
| Query= tetW_FWF624.PCC1F.w18 |
| Query= tetW_23-PCC1R.w21 |
| **Query= tetO_390.PCC1R** |
| Query= tetO_392.PCC1F |
| **Query= tetO_395.PCC1R** |
| Query= tetO_397.PCC1F |
| Query= tetO_397.PCC1R |
| **Query= tetO_398.PCC1F** |
| Query= tetO_403.PCC1R |
| **Query= tetO_405.PCC1F** |
| Query= tetO_411.PCC1F |
| Query= tetO_415.PCC1F |
| Query= tetO_419.PCC1R |
| Query= tetO_421.PCC1R |
| Query= tetO_429.PCC1F |
| Query= tetO_432.PCC1R |
| **Query= tetO_435.PCC1R** |
| **Query= tetO_436_32-T7pro** |
| Query= tetO_443.PCC1F |
| Query= tetO_444.PCC1F |
| Query= tetO_445.PCC1F |
| Query= tetO_452.PCC1R |
| Query= tetO_459.PCC1F |
| **Query= tetO_464.PCC1F** |
| Query= tetO_464.PCC1R |
| Query= tetO_482_24-T7pro |
| Query= tetO_486.PCC1F |
| **Query= tetO_486.PCC1R** |
| Query= tetO_487.PCC1F |
| Query= tetO_491.PCC1F |
| Query= tetO_492.PCC1R |
| Query= tetO_493.PCC1R |
| Query= tetO_494.PCC1F |
| **Query= tetO_494.PCC1R** |
| Query= tetO_501.PCC1R |
| Query= tetO_512_49-PCC1R |
| Query= tetO_515.PCC1F |
| Query= tetO_516.PCC1F |
| Query= tetO_517.PCC1F |
| Query= tetO_527.PCC1R |
| Query= tetO_530.PCC1R |
| Query= tetO_532.PCC1F |
| **Query= tetO_533.PCC1F** |
| **Query= tetO_533.PCC1R** |
| Query= tetO_539_29-PCC1R |
| Query= tetO_554.PCC1F |
| Query= tetO_554.PCC1R |
| Query= tetO_555.PCC1R |
| Query= tetO_556.PCC1F |
| Query= tetO_556.PCC1F |
| Query= tetO_556_36-PCC1R |
| Query= tetO_562.PCC1R |
| **Query= tetO_566.PCC1R** |
| Query= tetO_571.PCC1F |
| Query= tetO_572.PCC1R |
| Query= tetO_579.PCC1F |
| Query= tetO_588.PCC1F |
| Query= tetO_589.PCC1R |
| Query= tetO_603.PCC1F |
| **Query= tetO_605.PCC1F** |
| Query= tetO_608.PCC1R |
| Query= tetO_615.PCC1R |
| Query= tetO_611.PCC1F |
| **Query= tetO_619.PCC1R** |
| **Query= tetO_620.PCC1F** |
| **Query= tetO_620.PCC1R** |
| Query= tetO_622.PCC1F |
| Query= tetO_623.PCC1R |
| Query= tetO_629.PCC1F |
| Query= tetO_630.PCC1R |
| Query= tetO_635.PCC1F |
| **Query= tetO_637.PCC1F** |
| Query= tetO_638.PCC1F |
| Query= tetO_638.PCC1R |
| Query= tetO_646.PCC1F |
| **Query= tetO_647.PCC1R** |
| Query= tetO_648.PCC1F |
| Query= tetO_648.PCC1R |
| Query= tetO_655.PCC1F |
| **Query= tetO_663.PCC1R** |
| **Query= tetO_672_48-T7pro** |
| Query= tetO_676.PCC1R |
| **Query= tetO_683.PCC1F** |
| Query= tetO_684.PCC1R |
| Query= tetO_685.PCC1R |
| Query= tetO_691.PCC1F |
| Query= tetO_694.PCC1R |
| Query= tetO_698.PCC1F |
| Query= tetO_699.PCC1F |
| **Query= tetO_709.PCC1F** |
| **Query= tetO_709.PCC1R** |
| Query= tetO_710_42-PCC1F |
| Query= tetO_722.PCC1F |
| Query= tetO_722.PCC1R |
| **Query= tetO_723.PCC1F** |
| Query= tetO_727.PCC1R |
| Query= tetO_733.PCC1R |
| Query= tetO_734.PCC1R |
| Query= tetO_735.PCC1F |
| Query= tetO_738.PCC1F |
| **Query= tetO_739.PCC1F** |
| Query= tetO_740.PCC1R |
| Query= tetO_743.PCC1R |
| Query= tetO_746.PCC1F |
| **Query= tetO_748.PCC1R** |
| **Query= tetO_750.PCC1R** |
| Query= tetR_404.PCC1F |
| **Query= tetR_412.PCC1F** |
| Query= tetR_428.PCC1R |
| Query= tetR_460.PCC1R |
| Query= tetR_463.PCC1F |
| Query= tetR_484.PCC1F |
| Query= tetR_484.PCC1R |
| Query= tetR_495.PCC1R |
| Query= tetR_503.PCC1F |
| Query= tetR_504_60.T7pro |
| Query= tetR_541.PCC1R |
| Query= tetR_551_55.PCC1R |
| Query= tetR_584.PCC1F |
| Query= tetR_604.PCC1F |
| **Query= tetR_612.PCC1F** |
| **Query= tetR_632.PCC1R** |
| Query= tetR_636.PCC1F |
| Query= tetR_659.PCC1F |
| Query= tetR_659.PCC1R |
| Query= tetR_679.PCC1R |
| Query= tetR_686.PCC1F |
| Query= tetO_706.PCC1F |
| Query= tetR_718.PCC1F |
| Query= tetR_725_53.PCC1R |
