# Supplementary material for: The Gut as Reservoir of Antibiotic Resistance: Microbial Diversity of Tetracycline Resistance in Mother and Infant
Source: PLoS One. 2011 Jun 28;6(6):e21644. doi: 10.1371/journal.pone.0021644 (PMC3125294; doi:10.1371/journal.pone.0021644)
Supplement: Table S6 — Review of taxonomical classification of BLASTX hit sequences used by MEGAN to assign reads at species level in the infant Tcr metagenome. (DOCX) [file pone.0021644.s010.docx]

**Table S6.** Review of taxonomical classification of BLASTX hit sequences used by MEGAN to assign reads at species level in the infant Tc^r^ metagenome.

| Species, strain assigned by MEGAN | Accession no. | Reference PID | 16S | Notes | No. of reads assigned | Taxonomical evaluation |
| --- | --- | --- | --- | --- | --- | --- |
| *Streptococcus infantarius* subsp. *infantarius* ATCC BAA-102 | ABJK02000015 | N.P | N.A | L33- and L32 50 ribosomal subunit was annotated and blasting these ORFs against GenBank gave many highly similar or identical hits within the *Streptococcus* genus. | 3 | *Streptococcus* |

N.P. Not published

N.A. Not available
