# Supplementary material for: The Gut as Reservoir of Antibiotic Resistance: Microbial Diversity of Tetracycline Resistance in Mother and Infant
Source: PLoS One. 2011 Jun 28;6(6):e21644. doi: 10.1371/journal.pone.0021644 (PMC3125294; doi:10.1371/journal.pone.0021644)
Supplement: Table S7 — Review of taxonomical classification of BLASTX hit sequences used by MEGAN to assign reads at species level in the mother Tcr metagenome. (DOCX) [file pone.0021644.s011.docx]

**Table S7.** Review of taxonomical classification of BLASTX hit sequences used by MEGAN to assign reads at species level in the mother Tc^r^ metagenome.

| Species, strain assigned by MEGAN | Accession no. | Reference PID | 16S accession no. | Notes | No. of reads assigned | Taxonomical evaluation |
| --- | --- | --- | --- | --- | --- | --- |
| *Bacteroides pectinophilus* | ABVQ01000036 | N.P | [ABVQ01000036](http://www.ncbi.nlm.nih.gov/nuccore/ABVQ01000036) REGION: 352472..353992, DQ497993 | 16S was classified by Rdp classifier as unclassified Lachnospiraceae (98%) | 13 | Lachnospiaceae |
| *Bacteroides sp*. 3_1_33FAA | GG705230 | N.P. | N.A. | L28- and L33 50S are annotated. Blasting both ORFs against GenBank gives 3 and 1 identical/ highly similar hits within *Bacteroides* respectively and the best hit sequence is published. | 4 | *Bacteroides* |
| *Roseburia inulinivorans* DSM 16841  (or *Faecalibacterium prausnitzii* A2-165) | ACFY01000004 (or ACOP02000008) | N.P | Reference to AJ270473 (or AJ270469) | The best blastx hit used by MEGAN to assign the reads actually consisted of 2 identical sequences one classified as *Roseburia inulinivorans* DSM 16841 and one as *Faecalibacterium prausnitzii* A2-165. The former 16S (AJ270473) was classified by Rdp classifier as *Roseburia* (100%) and the latter 16S (AJ270469) was classified by Rdp classifier as *Faecalibacterium* (100%). Thus the lowest common ancestor (LCA) was Clostridiales. | 3 | Clostridiales |
| *Coprococcus comes* ATCC 27758 | ABVR01000046 | N.P. | N.A. | For all 4 reads the top 5 blastx hits belong to Clostridiales. | 4 | Clostridiales |
| *Dorea formicigenerans* ATCC 27755 | AAXA02000015 | N.P. | [AAXA02000015](http://www.ncbi.nlm.nih.gov/nuccore/AAXA02000015) REGION: 452491..454009 and Reference to L34619 | 16S was classified by Rdp classifier as *Dorea* (100%). Thus the classification as *Dorea* formicigenerans ATCC 27755 was correct. | 16 | *Dorea* |
| *Clostridium asparagiforme* DSM 15981 | ACCJ01000017 | N.P. | Reference to AJ582080 | 16S was classified by Rdp classifier as [unclassified_Clostridiales](http://rdp.cme.msu.edu/classifier/hierarchy.jsp?root=-212&depth=0&confidence=0.8). Thus the classification as *Clostridium asparagiforme* DSM 15981 appears to be correct. | 1 | *Clostridium* |
| *Clostridium leptum* DSM 753 | ABCB02000021 | N.P. | Reference to M59095 | 16S was classified by Rdp classifier as  "Ruminococcaceae" (100%) *Anaerotruncus* (86%). The LCA of *Clostridium leptum* DSM 753 and *Anaerotruncus* is Clostridiales. | 1 | Clostridiales |
| *Clostridium sp*. L2-50 | AAYW02000015 | N.P. | Reference to AJ270491 | 16S was assigned by Rdp classifier as *Coprococcus* (100%). The LCA of *Clostridium sp*. L2-50 and *Coprococcus* is Clostridiales. | 3 | Clostridiales |
| *Clostridium sp*. SS2/1 | [ABGC03000029](http://www.ncbi.nlm.nih.gov/nuccore/162702559) and [ABGC03000004](http://www.ncbi.nlm.nih.gov/nuccore/162702621) | N.P. | Reference to AY305319 | 16S was assigned by Rdp classifier to "Lachnospiraceae" (98%) *Anaerostipes* (82%). The LCA of *Clostridium sp*. SS2/1 and *Anaerostipes* is Clostridiales. | 3 | Clostridiales |
| *Clostridium scindens* ATCC 35704 | ABFY02000002 | N.P. | Reference to AF262238 | 16S was assigned by Rdp classifier as  Lachnospiraceae" (99%) *Dorea* (99%). The LCA of *Clostridium scindens* ATCC 35704 and *Dorea* is Clostridiales. | 1 | Clostridiales |
| *Eubacterium eligens* ATCC 27750 | CP001106 | [19321416](http://www.ncbi.nlm.nih.gov/pubmed/19321416) | N.A. | This sequence is published and the classification appeared to be correct. | 1 | *Eubacterium* |
| *Ruminococcus lactaris* ATCC 29176 | ABOU02000032 and [ABOU02000024](http://www.ncbi.nlm.nih.gov/nuccore/197298903) | N.P. | Reference to L76602 | 16S was assigned by Rdp classifier as  *Ruminococcus* (100%). Thus the classification was correct. | 4 | *Ruminococcus* |
| *Ruminococcus gnavus* ATCC 29149 (or *Blautia hansenii* DSM 20583) | AAYG02000006 (or  ABYU02000014) | N.P. | Reference to L76597 or M59114 | The best blastx hit used to by MEGAN to assign the read actually consisted of 2 identical sequences one classified as *Ruminococcus gnavus* ATCC 29149 and one as *Blautia hansenii* DSM 20583. The former 16S (L76597) was assigned by Rdp classifier as [unclassified_"Lachnospiraceae](http://rdp.cme.msu.edu/classifier/hierarchy.jsp?root=-2880&depth=0&confidence=0.8)” and the latter 16S (M59114) was assigned by Rdp classifier as *Blautia* (100%). Thus the LCA is these are Clostridiales. | 1 | Clostridiales |
| *Ruminococcus obeum* ATCC 29174 | AAVO02000002 | N.P. | Reference to L76601 | 16S was assigned by Rdp classifier as  *Blautia* (100%). The LCA of *Ruminococcus obeum* ATCC 29174 and *Blautia* is [Clostridiales](http://rdp.cme.msu.edu/classifier/hierarchy.jsp?root=211&depth=0&confidence=0.8). | 17 | [Clostridiales](http://rdp.cme.msu.edu/classifier/hierarchy.jsp?root=211&depth=0&confidence=0.8) |
| *Ruminococcus sp*. 5_1_39BFAA | GG696045 | N.P. | N.A. | The sequence has no 16S annotated or any reference to the taxonomical classification. Thus the read assignment is moved to Clostridiales. | 1 | Clostridiales |
| *Faecalibacterium prausnitzii* M21/2 | ABED02000024 | N.P. | Reference to AY305307 | 16S was assigned by Rdp classifier as *Faecalibacterium* (100%). Thus the initial classification was correct. | 2 | *Faecalibacterium* |

N.P. Not published

N.A. Not available

Rdp classifier: Ribosomal database project classifier (<http://rdp.cme.msu.edu/classifier/classifier.jsp>)

LCA: Lowest common ancestor
